# Activity of Amorphous NiB/SiO_2_ in Hydrotreating Model Reactions: Hydrodesulfurization of 4,6-DMDBT

**DOI:** 10.1021/acsomega.5c04966

**Published:** 2025-08-20

**Authors:** Marek Lewandowski, Rafał Janus, Mariusz Wądrzyk, Karolina Jaroszewska, Kamila Zaborowska

**Affiliations:** † Faculty of Energy and Fuels, AGH University of Krakow, 30 Mickiewicza, PL-30059 Kraków, Poland; ‡ Faculty of Chemistry, Wrocław University of Science and Technology, 7/9 Gdan’ska, PL-50344 Wrocław, Poland

## Abstract

For the first time, we examined the catalytic performance of a NiB/SiO_2_ catalyst with 10 wt % NiB in model hydrodesulfurization of 4,6-dimethyldibenzothiophene (4,6-DMDBT) also together with a competing nitrogen compound, that is, carbazole. The NiB/SiO_2_ catalyst (fresh, reduced, and spent) was characterized using the following techniques: N_2_ sorption, ICP, XRD, CO chemisorption, XPS, and elemental analysis. The results of XRD, XPS, and elemental analysis indicated the partial decomposition of the NiB phase into metallic nickel (accompanied by boron atoms) and partial sulfidation into Ni_3_S_2_ species under reaction conditions. FT-IR spectra have shown the formation of −SH moieties on the NiB/SiO_2_ surface. As revealed in the distribution of the products, regardless of the reaction mode (in the absence or presence of carbazole), the dominant conversion pathway for 4,6-DMDBT was its hydrogenation (HYD route) to 3-(3′-methylcyclohexyl)-toluene (MCHT). The efficiency of dimethylbiphenyl (3,3′-DMBPh) formation was low and was weakly dependent on contact time. Furthermore, it was found that the donation of electrons from B to Ni species tuned the electron structure, not only enhancing the selectivity of the HYD pathway but also improving the thioresistance of the catalyst. As a result of the strong adsorption of carbazole on the catalytic centers, the phenomenon of hindering the transformation of 4,6-DMDBT is observed. Nevertheless, the NiB/SiO_2_ catalyst was found to be active in the HDS reaction for about 100 h.

## Graphical abstract

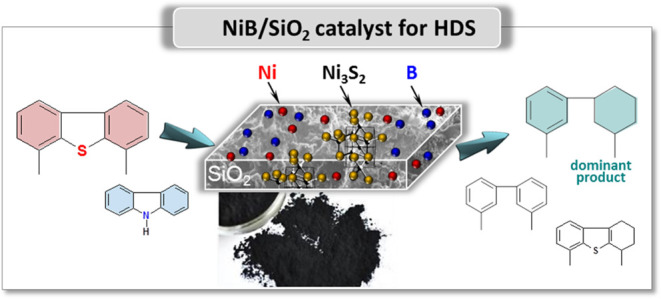

## 1 Introduction

### 1.1 Sulfur Content Requirements

Hydrodesulfurization (HDS) of fuel fractions is a complex environmental and technological issue. In recent years, more rigorous environmental restrictions have accelerated the research for profound improvements in HDS catalysts designed for manufacturing of ultraclean fuels.^1^ Sulfur in fuels, upon combustion, is released in the form of sulfur oxide (SO_*x*_) gases and sulfate particulate matter, which can poison the vehicle catalyst, deteriorate engine performance, contribute to environmental pollution, and affect living organisms. The heavy grades of crude oil extracted today are also a problem due to the high content of compounds containing sulfur and nitrogen, as well as polycyclic aromatic hydrocarbons and metals.^2^ Furthermore, to meet the global demand for motor fuels, waste liquefaction processes, such as the pyrolysis of used tires, are becoming increasingly common for high-quality fuels that require the removal of hazardous sulfur compounds.^3^ For that reason, interest has grown over the past few years in seeking efficient catalysts for the hydrotreatment of the mentioned heavy fractions. Since 2009, regulations have required the removal of sulfur in transportation fuels to a very low level, that is, up to 10 ppm.^4^ In the future, the sulfur content is also expected to decrease even further below 1 ppm, especially for fuel cell applications.^4^ It should be mentioned, however, that sulfur compounds exquisitely improve the lubricity of diesel fuels. Owing to this, in order to compensate for the lack of lubricity of ultradeeply desulfurized fuels, suitable lubricity improvers have to be used.

### 1.2 Deep Hydrotreating

The industrial HDS process is carried out in two stages on sulfided Mo- or W-based catalysts promoted with Ni or Co and loaded over the γ-Al_2_O_3_ carrier. In the first stage, most of the sulfur compounds are removed, while in the second one, the refractory S-compounds (those with steric hindrances) are removed and part of the aromatics are hydrogenated. The sulfur content remaining after the first hydrotreating stage ranges from 250 to 300 ppm, and the nitrogen content reaches about 100 ppm.^5^ The presence of impurities containing refractory S and N forces existing technologies to perform the second HDS stage, i.e., ultradeep HDS, which requires the use of high-priced operating regimes and catalysts.^6^ Such impurities are mainly dibenzothiophene (DBT) and carbazole and their alkyl derivatives. The cause of the refractory properties of dialkyldibenzothiophenes (mainly 4,6-dimethyldibenzothiophene; 4,6-DMDBT) is linked to the steric obstacle in the elimination reaction involved in C–S bond breaking. HDS of 4,6-DMDBT occurs through two main routes, i.e., hydrogenation (HYD) and direct desulfurization (DDS).^7^ It is pertinent to mention that the favored reaction path depends on the conditions and the catalyst used. In traditional CoMo or NiMo catalysts, C–S bond cleavage is preferred using the MoS_2_ active phase, primarily via the DDS route, where these catalysts demonstrate their selectivity. On the other hand, supported Pd–Pt noble metal catalysts are characterized by high activity in the HDS reaction and high selectivity toward the HYD pathway. The HDS reaction is kinetically controlled, and the HYD pathway is preferred.^8,9^ The HYD pathway appears to be more favorable for the removal of the sulfur atom from 4,6-DMDBT due to the steric hindering effect. However, the DDS pathway is often suppressed for the 4,6-DMDBT molecule, which cannot adsorb on the catalyst active sites. Another aspect of the reactivity of the hard-to-remove sulfur derivatives concerns the inhibiting effect of nitrogen compounds. N-containing molecules, such as carbazole and its alkyl derivatives, exhibit a very strong inhibitory action on deep HDS and self-inhibiting hydrodenitrogenation reactions (HDN) due to the competing adsorption of sulfur- and nitrogen-containing molecules over catalytic centers.^10^ Furthermore, residual nitrogen compounds (after the hydrotreatment process) would not only inhibit ultradeep HDS but also poison acid catalysts during the subsequent steps of fluid catalytic cracking and hydrocracking.^11^ Moreover, the adsorption mode of polynuclear aromatic compounds has been reported to be similar to that of carbazole and, therefore, these molecules are expected to inhibit HDS.^12^ The most active HDS catalysts are based on two different approaches to the catalyst design. In 2001, AkzoNobel, ExxonMobil, and Nippon Ketjen developed unsupported catalysts based on NEBULA technology. These catalysts have shown HDS and HDN activities superior to those of conventional alumina-supported hydrotreating catalysts.^10,13^ This new generation of catalysts is based on a completely different concept of bulk-like materials with the highest HYD function. They are made up of unsupported ternary NiMoW systems with a porous structure formed through decomposition of the metal precursors. Another possibility is to use the BRIM Ni­(Co)­Mo/γ-Al_2_O_3_ catalyst developed by Haldor Topsøe and published by Weise et. al.^14^ This catalyst exhibits a metallic character due to the formation of the Mo­(W)­S_2_ phase, which has numerous “brim sites” which are completely sulfur-coordinated centers. These sites are involved in adsorption, C–S bond breaking, and hydrogenation. Hence, the NiMo­(CoMo) catalysts display HYD activity and an enhanced rate of DDS reaction routes. The presented catalytic systems show great potential for commercial applications because of their exquisite hydrotreating performance. Nevertheless, they are still limited by the high metal content and the resulting high costs. It is clearly visible that the challenge for HDS technology is still the development of highly active catalysts with an acceptable level of production. To overcome these drawbacks, researchers have intensively investigated catalysts supporting various materials and catalysts with various active phase compositions. Furthermore, much effort has been put into improving the properties of the HDS catalyst in order to drive the HDS reaction toward one of the pathways as a result of HDS selectivity, which has a crucial impact on the process costs and the ultimate product quality. On the one hand, the DDS route consumes a lower amount of H_2_ than HYD, thus being more economic. On the other hand, the HYD pathway provides a fraction with a lower number of aromatic rings. In recent decades, amorphous alloys such as transition metal borides, owing to their unique long-range disordered and short-range ordered structures, have received growing interest in numerous application areas, including catalysis.^15^

### 1.3 Nickel Boride—NiB

In view of their good performance in hydrogenation and resistance to sulfur poisoning, amorphous transition metal boride alloys could be effective HDS catalysts.^16,17^ NiB is known as the highest active metal boride-based catalysts. The high resistance of NiB to sulfur poisoning is an essential feature, taking into account the quality of the raw products subjected to the process. Luo et al.^18^ have studied the adsorption of sulfur on NiB alloys using density functional theory calculations. According to their results, sulfur prefers bonding to boron and not to nickel in the alloy NiB catalyst, which leads to protection of the active nickel sites from deactivation by sulfur compounds. These findings are fully consistent with those reported previously by others^19,20^ who postulate that in NiB alloys, sulfur is irreversibly adsorbed on elemental boron. Meanwhile, Lewandowski carried out the first HDS investigation of 4,6-DMDBT on the NiB alloy together with an HDN reaction of the nitrogen compound (carbazole).^21,22^ It has been revealed that during HDN/HDS reactions, the phases of Ni_3_S_2_, Ni_3_B, and Ni^0^ are partially formed. The author ascribed the activity of this catalyst mainly to the presence of Ni^0^ in the environment of well-dispersed boron species and/or Ni_3_B phases. The effect of boron on the electronic state of nickel in the NiB alloy has been a subject of interest to many authors.^18–20^ Interactions of this type can substantially affect the magnetic or catalytic properties of the alloy. It is commonly accepted that the strong catalytic ability of NiB catalysts is related to electron donation from boron to the accompanying Ni species in the alloy. Some of the boron electrons occupy the partly empty orbital d of nickel, making it electron-deficient, while the nickel atom is enriched in electrons. As a result, boron shows a greater affinity for available free electron pairs, e.g., on sulfur or oxygen atoms, than nickel and protects the metal from sulfur poisoning or oxidation. On the other hand, electron-enriched nickel shows an affinity for unsaturated bonds; therefore, it becomes active in hydrogenation reactions. Also recently, Linares and Brunet^8,9^ studied the NiCo double boride in the HDS of DBT reaction. These catalysts showed a preferred hydrogenation path based on the higher HYD/DDS ratio compared to classical HDS of DBT. Moreover, authors postulated similar behavior of this catalyst to the noble metal-based catalysts.

It also became interesting to answer the question of how a supported catalyst would behave under such conditions. The role of the support is, among others, to ensure, through appropriate interactions between the active phase and the support, the optimal concentration and composition of the active phase and its dispersion and morphology. Because of the achievement of appropriate properties, it is possible to obtain a catalyst with a lower content of the active phase while maintaining high activity. As a result, the overall cost of catalyst synthesis is reduced. In addition, because of the weak thermal stability and low specific surface area of amorphous alloy materials, depositing them on a high surface support is one of the solutions for improving these features. Therefore, selection of the support for NiB catalyst tuning of the active phase–support interaction is of great importance to achieve the proper dispersion of the active species. Data from the literature indicate that SiO_2_ or SiO_2_-containing materials are the most commonly used supports for boride catalysts in various hydrogenation reactions.^8,9,16,23,24^

Here, we propose an original approach to the synthesis of a supported NiB/SiO_2_ catalyst for the HDS/HDN reaction. To the best of our knowledge, no work considering such a catalytic system for hydrotreatment processes has been reported. The main purpose of this investigation is to examine the properties and activity of the NiB/SiO_2_ catalyst in the HDS reaction and the effect of the N-containing molecule (carbazole) on this reaction. Furthermore, there is still a significant gap in the fundamental identification of the active species in the NiB catalyst involved in the reaction. Therefore, this work also aims to unravel the nature of the active species present in NiB/SiO_2_. We also discuss the significance of catalyst activation in the active phase composition and the effect of NiB/SiO_2_ features on its catalytic activity. We believe the present research makes a significant contribution to the design of catalysts for the second stage of hydrotreating processes.

## 2 Experimental Section

### 2.1 Materials

Chemicals used for the preparation of the NiB/SiO_2_ catalyst: ethanol (Avantor Performance Materials Poland, 99.9%), nickel­(II) chloride hexahydrate (NiCl_2_ × 6H_2_O, Sigma-Aldrich, 99.9%), and potassium borohydride (KBH_4_, Sigma-Aldrich, 98%). The silica support was supplied by Sigma-Aldrich. 4,6-Dimethyldibenzothiophene (4,6-DMDBT, Acros Organics 95%) and carbazole (Sigma-Aldrich 95%) were used as model compounds for the HDS/HDN reactions. Silicon carbide (SiC, Fluka) was used as an inert material for the dilution of the catalyst prior to the catalytic run. All aqueous solutions were freshly prepared by using deionized water.

### 2.2 Preparation of NiB/SiO_2_ Catalysts

The NiB/SiO_2_ catalyst was prepared according to the procedure reported by Zhang et al.^25^ First, the support was impregnated with an aqueous solution of NiCl_2_ and dried at 120 °C for 12 h. The obtained catalyst precursor was then reduced by a dropwise addition of 0.2 M KBH_4_ solution. The initial Ni/B ratio was 2.5 to 1. This allowed for a total reduction of the Ni^2+^ ions. The prepared sample was then washed with deionized water until the Cl^–^ ions had disappeared (this was determined by the reaction of Cl^–^ ions with AgNO_3_). Finally, the sample was washed with ethanol.

### 2.3 Characterization

#### 2.3.1 Textural Properties of Catalysts

Textural parameters of the catalysts were determined by low-temperature physisorption of nitrogen at −196 °C. The isotherms were recorded on a Micromeritics instrument ASAP2020. Before the measurements, the samples were evacuated as follows: (i) Evacuation Phase: temperature ramp rate: 10.0 °C min^–1^, target temperature: 90 °C, evacuation rate: 10 mmHg s^–1^, evacuation time: 60 min, (ii) heating phase: ramp rate: 10.0 °C min^–1^, hold temperature: 300 °C, hold time: 10 h. Specific surface areas (*S*_BET_) were calculated based on the multipoint Brunauer–Emmett–Teller (BET) model (*p*/*p*_0_ = 0.05–0.20). External surface areas (*S*_ext_) and micropore volumes (*V*_μ_) were assessed based on α_s_ plots (macroporous silica LiChrospher Si-1000 was employed as the reference). The *t*-plot model was employed for calculations of the micropore surface (*p*/*p*_0_ = 0.05–0.20). Single-point total pore volumes (*V*_t_) were extracted from the adsorption branches of isotherms using the respective data points at *p*/*p*_0_ = 0.98. The pore size distributions of the catalysts (*D*_NLDFT_) were calculated by applying the nonlocal density functional theory kernel (NLDFT; adsorption branch; cylindrical pore symmetry was assumed).

#### 2.3.2 Dynamic CO Titration-Pulsed Technique

CO uptake is a classical technique that is used to titrate metallic sites. CO uptake was carried out in situ, that is, in the quartz synthesis reactor, without exposure of the fresh NiB alloy supported on SiO_2_ to air. Pulses of a known amount of CO (17 μmol) were injected at regular time intervals on the sample at room temperature in flowing He (40 μL min^–1^) purified by an O_2_ trap (Oxysorb, Messer Griesheim). After each injection, the quantity of CO not chemisorbed was measured by using a conventional TCD device. The injections were continued until the surface was fully saturated with the adsorptive. The amount of CO chemisorbed was determined as a number of micromoles per gram of the catalyst.

#### 2.3.3 X-ray Diffraction

Structural characterization of NiB catalysts before and after reactions was performed by using X-ray diffraction (XRD) using a Siemens D-500 automatic apparatus with Cu Kα monochromatized radiation. The amount of amorphous phase and crystallinity of the nickel boride have been estimated from the intensity of all reflections in the angular range from 10° to 90°. The diffractograms were compared with those classified by the JCPDS index. The average sizes of the zerovalent nickel and Ni_3_S_2_ crystallites in the spent catalyst were evaluated based on the Scherrer formula from the line broadening of the most intensive Ni^0^ and Ni_3_S_2_ peaks at 2θ of 44.42° and 31.13°, respectively, using the K shape factor of 0.9.

#### 2.3.4 Elemental Chemical Analysis

Elemental analysis for nickel and boron compositions was performed with the ICP method with the ULTIMA 2 spectrometer. The sample was mineralized with a Multiwave 3000 mineralizer.

#### 2.3.5 Elemental CHNS Analysis after Reactions

The elemental analysis of the NiB/SiO_2_ catalyst (C, H, N, and S elements) after the HDS reaction was performed by using the EuroVector CHNS analyzer, model EA3100. The sample was weighed in tin capsules and put in an oxidation/reduction reactor held at a temperature of 900 to 1000 °C. Oxidation of the tin capsule and sample caused a local temperature increase in the reactor up to 1800 °C in a few seconds. At such a high temperature, inorganic and organic compounds are oxidized to gaseous products (CO_2_, H_2_O, SO_2_, and NO_*x*_). Nitric oxides are reduced to elemental N_2_. The analyzed gases are separated in a chromatographic column and quantitatively determined using a highly selective thermoconductive detector (TCD).

#### 2.3.6 X-ray Photoelectron Spectroscopy

XPS analysis was performed on the catalyst reduced with hydrogen samples for 2 h at 450 °C and a hydrogen flow of 60 cm^3^ min^−1^ and a sample of the spent catalyst after the HDS/HDN process after about 100 h time-on-stream.

The chemical states of the superficial elements of the catalysts were studied by using X-ray photoelectron spectroscopy (XPS). A VSW (Vacuum Systems Workshop Ltd.) instrument with a concentric hemispherical electron analyzer (radius of 150 mm) and a two-plate 18-channel detector (model Galileo) was operated in a fixed analyzer transmission (FAT) mode with a constant pass energy of electrons equal to 22.5 eV. Samples were exposed to the Kα Mg (1253.6 eV) X-ray radiation emitted by an X-ray lamp working at a power of 208 W (13 kV acceleration voltage, 16 mA emission current). The residual vacuum pressure during the analyses was less than 5×10^–8^ mbar. The position of the C 1s line ascribed to the C–H bond was established at 284.8 eV to calibrate the energy scale.

#### 2.3.7 Fourier Transform Infrared Spectroscopy

Fourier transform infrared (FT-IR) spectra of the catalysts (300 scans each) were collected in the mid-infrared wavenumber range (650–4000 cm^–1^) at a resolution of 4 cm^–1^ using a Nicolet iS5 FT-IR spectrometer (Thermo Scientific) equipped with a DTGS detector and a diffuse reflectance (DRIFT) device (EasiDiff-Pike Technologies). Prior to the measurements, the catalysts were diluted with potassium bromide (KBr, 99+%, Acros Organics) at 5 wt % and gently milled in an agate mortar.

### 2.4 Catalytic Test

The HDS alone and simultaneous HDS/HDN reactions were performed in a high-pressure fixed-bed microreactor operating in a flow system. The catalyst (0.8 g) was mixed with SiC in a mass ratio of 1/5 catalyst to SiC. The hydrogen flow was set in the range of 60 to 360 cm^3^ min^–1^. The conversion of 4,6-DMDBT and the degree of HDS of 4,6-DMDBT were calculated as follows:

1 $$conversion\;of\;4,6‐DMDBT=\frac{S_{S}}{S_{S}+S_{4,6‐DMDBT}}\times 100\%$$

2 $$S\;removal\;rate=\frac{S_{H}}{S_{S}+S_{4,6‐DMDBT}}\times 100\%$$

where *S*_S_ refers to the sum of the molar percentages of all products formed after the catalytic transformation of 4,6-DMDBT (including S-containing compounds). *S*_4,6‑DMDBT_ means that the molar percentage of 4,6-DMDBT is still present after the reaction. *S*_H_ is the sum of the molar % of all products that do not contain sulfur, formed after the catalytic transformation of 4,6-DMDBT. The liquid products of the reactions were collected at 1 h intervals in the condenser thermostated at 15 °C. Finally, these products were quantitively analyzed by a GC (HP 4890) equipped with a capillary column (HP1, 30 m × 0.25 mm × 0.25 μm) and a flame ionization detector (FID). The qualitative identification of the products was performed by means of GC–MS analysis. An Agilent Technologies chromatograph (GC model 5890B) with a capillary column (DB17, 30 m × 0.25 mm × 0.25 μm) coupled to an MS model 5977A was used. The HDS reaction of 4,6-DMDBT was performed separately and in the presence of carbazole at 350 °C under a total pressure of 6.0 MPa with a volume H_2_/feed ratio of 600. The liquid feedstock contained 0.17 wt % of 4,6-DMDBT (300 ppm of S) or 0.17 wt % of 4,6-DMDBT and 0.08 wt % of carbazole (100 ppm of N) dissolved in *o*-xylene. Before starting the catalytic tests, each catalyst was heated in situ at 350 °C for 2 h in a pure dihydrogen atmosphere. The whole catalytic run was conducted for about 100 h. After the first 75 h, the initial operating conditions were reset to check again the activity of the catalyst. The conversion of solvent was checked for all catalysts. It was observed that practically no conversion of the solvent took place under the operating conditions. The catalysts after the reactions were collected and stored in solvent (i.e., *o*-xylene) for further characterization. Contact time (*t*_C_(*s*)) was defined as follows:

3 $$t_{C}\left(s\right)=\frac{catalyst\;volume\;\left({cm}^{3}\right)}{\left(H_{2}\;flow+feed\;flow\right)\;\left({cm}^{3}\;s^{-1}\right)}$$

The range of contact times tested ranged from 0.19 to 1.13 s for the HDS alone and HDS/HDN reactions, respectively.

## 3 Results

### 3.1 Textural Properties

The nitrogen isotherms of the pristine SiO_2_ support as well as the fresh and spent NiB/SiO_2_ catalysts (after HDS) are shown together with the respective pore size distributions in Figure 1. The calculated textural properties are summarized in Table 1.

**Figure 1:**
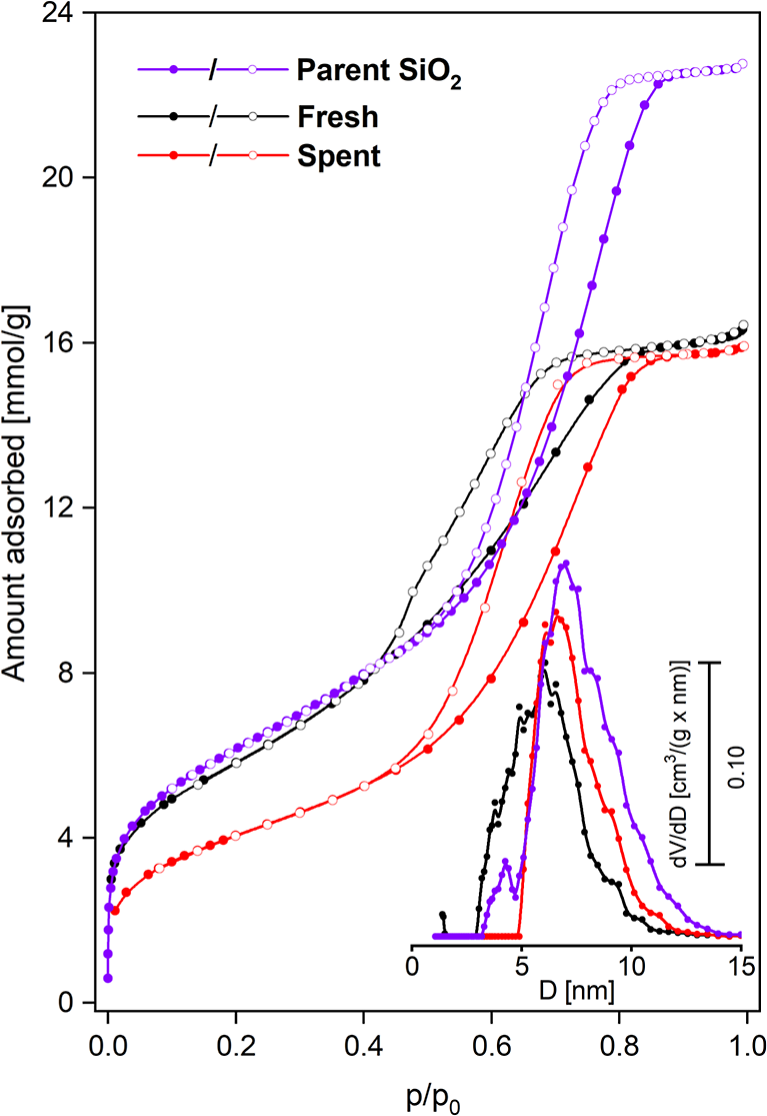
Nitrogen adsorption–desorption isotherms (open and closed symbols, respectively) recorded for the parent SiO_2_ and fresh and spent NiB/SiO_2_ catalysts (red—spent catalyst after approximately 100 h time-on-stream HDS/HDN reaction). The respective pore size distributions (NLDFT model) are displayed in the inset.

**Table 1:** Textural Parameters of the Parent Silica Support and Fresh and Spent NiB/SiO_2_ Catalysts (Spent Catalyst, i.e., after About 100 h of the HDS/HDN Process)

sample	*S*_BET_ (m^2^ g^–1^)	*S*_ext_ (m^2^ g^–1^)	*S*_μ_ ^a^ (m^2^ g^–1^)	*V*_T_ ^b^ (cm^3^ g^–1^)	*V*_μ_ ^c^ (cm^3^ g^–1^)	*D*_NLDFT_ ^d^ (nm)
parent SiO_2_	501	43	<10	0.79	0.00	7.0
NiB/SiO_2_ fresh	471	43	21	0.56	0.03	6.1
NiB/SiO_2_ spent	331	15	<10	0.55	0.01	6.6

a Surface of micropores from *t*-plot.

b Total pore volume from s–p.

c Micropore volume from α_s_.

d Pore diameter (NLDFT method).

According to the IUPAC classification,^26^ all isotherms can be classified as type IV­(a) with delayed adsorptive condensation in open-ended cylindrical mesopores accompanied by hysteresis loops. Interestingly, the hysteresis loops for the starting support and spent catalyst are purely H1 type disclosing the presence of mesopores uniform in size. Meanwhile, the hysteresis for the fresh catalyst resembles to some extent the behavior of H_2_(*b*) type (with a closure point at *p*/*p*_0_ ≈ 0.4). This is indicative of the effect of partial blocking of mesopores by the clusters of the fresh active phase; however, the necks of these pores are relatively broad. With this, one may infer that the active phase undergoes redistribution upon the HDS process. Indeed, taking into account the sulfidation of the NiB alloy supported on SiO_2_ and its coalescence, additionally accompanied by the coking effect, this scenario seems to be likely. Consequently, the spent material features a more homogeneous dispersion of the active phase. As a result, it reveals a narrower pore size distribution slightly shifted (by 0.5 nm) toward broader pores, resembling the pristine support (see Figure 1).

The pristine silica support reveals the specific surface area of 501 m^2^ g^–1^ with 43 m^2^ g^–1^ of the external surface area, the total pore volume of 0.79 cm^3^ g^–1^, and the pore size distribution centered at 7.0 nm. *S*_BET_ of the fresh NiB alloy supported on SiO_2_ was found to be 471 m^2^ g^–1^ (i.e., 6% decrease compared with the parent support) with 43 m^2^ g^–1^ of the external surface and 21 m^2^ g^–1^ of the micropore surface. It also shows a 29% drop in the total pore volume. The relatively high surface is mainly due to the well-developed surface of the support itself (also presented in Table 1). The *S*_BET_ decrease of the spent catalyst by ca. 30% with regard to the fresh one (i.e., 331 m^2^ g^–1^) is related mainly to the aforementioned superficial deposition of coke on the surface (as shown by elemental analysis) and the coalescence process of the active phase (NiB) as well as its partial sulfidation (formation of Ni_3_S_2_ as discussed below). This effect occurs primarily in the narrowest pores, and it is evident when considering a roughly 50% drop in the surface of the micropore and a 1/3 decrease in the volume of the micropore, while the total pore volume remains almost unchanged. It is pertinent to mention that the deposition of coke also occurs on the external surface of the catalyst’s grains (see the 2/3 drop in the external surface). However, both the specific surface area and the pore volume of the spent catalyst remained relatively high.

### 3.2 CO Chemisorption

The experimental CO uptake of the sample of the NiB alloy supported on SiO_2_ (after in situ heating at 350 °C for 8 h in the H_2_ atmosphere) was 456 μmol g^–1^. The stoichiometry factor for CO chemisorption on Ni metal is 2 (i.e., 1 CO molecule per 2 atoms of Ni—bridge type CO molecularity adsorption). Therefore, as calculated, there is 912 μmol of Ni atoms per one gram on the surface of the NiB alloy supported on SiO_2_, which is equal to 1.17 × 10^18^ atoms of Ni per 1 m^2^ of the catalyst specific surface. CO adsorption on nickel is much more complex, and its stoichiometry changes depending on the measurement pressure, temperature, metal crystallite size, and its amount on the support surface. The formation of nickel carbonyl and significant amounts of chemical and physical adsorption of CO on the support caused additional complications. Therefore, we believe that the obtained high CO chemisorption results do not reflect the actual surface condition. It should be assumed that the surface area of the nickel metal phase is much smaller than it results from stoichiometric calculations.^27,28^

### 3.3 Elemental Analysis

The quantitative composition of the freshly prepared NiB/SiO_2_ catalyst, calculated based on elemental analysis, was as follows: 102 mg/g of nickel and 6.9 mg/g of boron, which correspond to the stoichiometric ratio of Ni/B = 2.71:1. In the synthesized catalyst, the molar ratio of nickel to boron equaled nearly 3 to 1, which is typical for such systems, according to the other authors.^29^

The elemental CHNS analysis of the catalyst after HDS reaction of 4,6-DMDBT is presented in Table 2. Additionally, the nickel content in the spent catalyst was also analyzed, which was 9.6 wt %. It is slightly lower than the Ni content in the fresh catalyst (10.2%). The difference is caused by the presence of carbon deposits on the surface of the used catalyst (as “ballast”), which causes the determined concentration of Ni and B content to be slightly lower. Based on these results (elemental analysis and Ni content in the spent catalyst), it was calculated that only about 54.3% of Ni was transformed into Ni_3_S_2_ during the catalytic tests.

**Table 2:** Elemental Analysis for Spent NiB/SiO_2_ Catalysts (after About 100 h of the HDS and HDS/HDN Process)

sample	C (wt %)	H (wt %)	S (wt %)	N (wt %)
NiB/SiO_2_ after HDS	1.36	0.62	1.90	
NiB/SiO_2_ after HDS/HDN	2.67	1.11	1.81	0.15

Based on the results of the elemental analysis of CHNS and the ICP results of the Ni and B contents in the catalysts, Table 3 shows the calculations of the Ni/B, Ni/S, Ni/C, and Ni/N molar ratios. No loss of nickel or boron from the catalyst was observed (ICP analysis).

**Table 3:** Molar Ratios of Ni to Elements B, C, N, and S for Fresh and Spent Catalysts

sample	Ni/B	Ni/S	Ni/C	Ni/N
NiB/SiO_2_ fresh	2.71:1			
NiB/SiO_2_ after HDS	2.66:1	2.71:1	1.45:1	
NiB/SiO_2_ after HDS/HDN	2.64:1	2.80:1	1.40:1	14.7:1

The molar ratio of Ni to B is practically constant, as for the fresh catalyst (Table 3). Similarly, for the catalysts after HDS and HDS/HDN processes, the molar ratios of Ni to C and S are comparable.

### 3.4 X-ray Diffraction

The XRD patterns of the pristine SiO_2_ support and supported NiB catalysts are shown in Figure 2. For the silica support (Figure 2a), the typical broad diffraction peak was found at around 2θ = 22°, which is characteristic of amorphous SiO_2_ (JCPDS 36-1451).^30^ The degree of crystallization of NiB species under the reaction conditions exerts a great influence on the catalytic activity of NiB/SiO_2_. Therefore, to follow the active phase changes in the NiB/SiO_2_ catalyst under activation and reaction conditions, the samples (at different treatment stages) were analyzed by XRD. In Figure 2, three of the NiB/SiO_2_ catalyst patterns are demonstrated, i.e., for a fresh sample (Figure 2b), for a NiB/SiO_2_ sample subjected to 8 h of heating in hydrogen flow at a temperature of 350 °C (Figure 2c), and after the HDS process (Figure 2d). As can be seen, all NiB/SiO_2_ samples show the signal at the 2θ angle centered around 22°, which originates from the amorphous SiO_2_ support. In the XRD pattern of the parent NiB/SiO_2_ sample (Figure 2b), a broad reflection is seen at 2θ = 44°. Considering the characterization of similar NiB-based catalysts,^31,32^ this broad reflection could be attributed to the amorphous NiB phase (JCPDS 06-0567). The diffractogram does not reveal any reflection of the crystalline structure of NiB, which points to the typical amorphous structure of the fresh catalyst. No significant diffraction signals revealing NiB species are observed, probably due to the low NiB content and its high dispersion on the SiO_2_ support.^33^ The XRD spectrum for the sample reduced ex situ at 350 °C is shown in Figure 2c. As can be inferred, small diffraction lines with 2θ at 51.8° and 76.4° have appeared. These reflections correspond to the Miller indices (2 0 0) and (2 2 0), respectively, which are assigned to metallic Ni^0^ species (JCPDS 01-071-3740).^34^ The result also shows that the crystallization of the NiB phase takes place to some extent because of the narrowing and sharpening of the peak at 2θ = 44°. This diffraction peak is indexable as a reflection (1 1 1), which is also assigned to metallic Ni^0^ species.^34^ However, the peak at 2θ = 44° is broadened to some extent compared to the crystalline pattern of pure Ni, hinting that there are some domains of amorphous alloys in the catalyst after thermal treatment. The pattern of the spent NiB/SiO_2_ sample (Figure 2d) is substantially different from that discussed above, and additional changes upon long-term treatment are detectable for a spent NiB/SiO_2_ catalyst. It has been observed that the amorphous structure of NiB/SiO_2_ underwent decomposition after the reaction and, except for a broad reflection around 2θ = 22° (silica support), the diffractogram features a set of sharp lines. And so, further decomposition of the NiB alloy brings small signals to appear positioned at 2θ of 21.75°, 31.10°, 37°, 50°, and 55°. These lines could be considered typical for the crystallographic phase of Ni_3_S_2_, specific for the low temperature structure of the Ni_3_S_2_ sulfide of the heazlewoodite phase type,^35^ and ascribed to the Miller indices of (1 0 1), (1 1 0), (0 0 3), (1 1 3), and (1 2 2), respectively (JCPDS 00-030-0863). The 2θ peak around 35° appearing in the XRD spectrum in Figure 2d is related to the presence of SiC in the catalyst sample after the HDS reaction. SiC was used as the catalyst diluent and was present in the spent sample because of the difficulty in accurately separating the catalyst from the SiC after its removal from the reactor.

**Figure 2:**
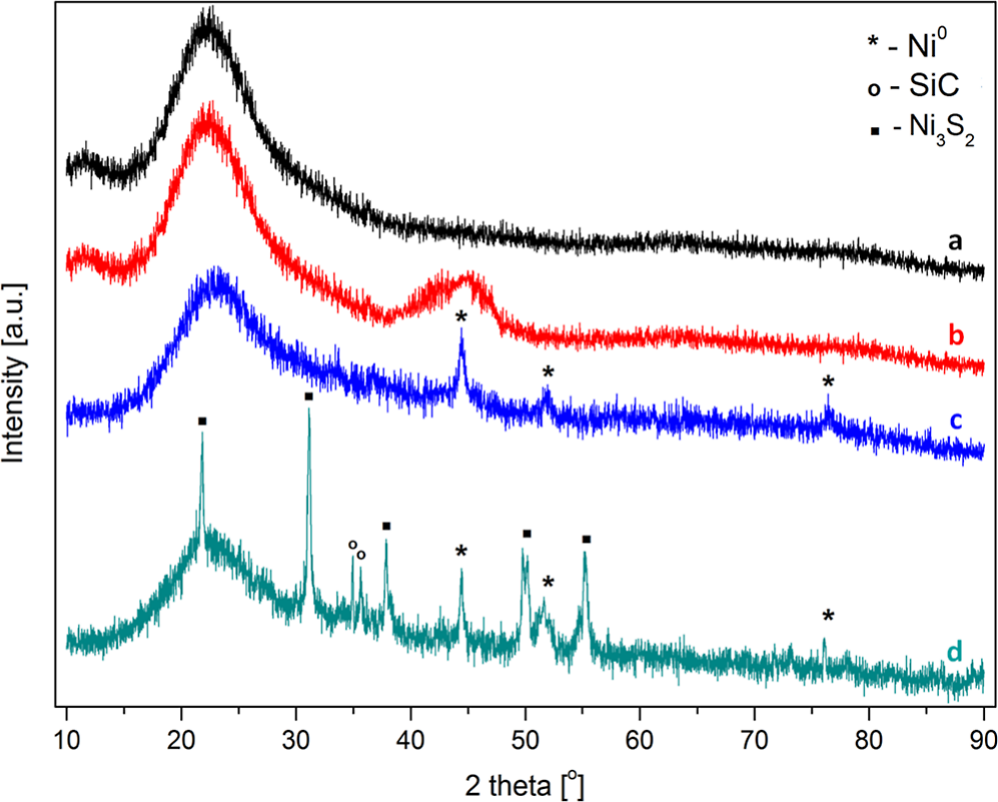
X-ray diffraction patterns of: (a) pristine SiO_2_ support, (b) fresh NiB/SiO_2_ catalyst, (c) NiB/SiO_2_ catalyst heated in H_2_ for 8 h at 350 °C, and (d) NiB/SiO_2_ catalyst after HDS/HDN reaction of 4,6-DMDBT.

Using the Scherrer formula, the average sizes of Ni and Ni_3_S_2_ crystallites on the catalyst after HDS were calculated from the XRD patterns. The average crystallite sizes for Ni and Ni_3_S_2_ were 21.7 and 26.9 nm, respectively. These values are consistent with those published elsewhere.^36,37^

### 3.5 X-ray Photoelectron Spectroscopy

One of the main goals of this research is to determine the variability of the chemical states of the SiO_2_-supported NiB catalyst undergoing hydrogen treatment and the HDS/HDN reaction. To provide information about the chemical composition and interfacial interactions, the H_2_-treated and spent catalysts were studied by XPS.

The presence of Ni, O, C, B, and Si elements was proven by the XPS scanning spectrum (Figure 3). For the reduced catalyst (Figure 3a), the following values were obtained: Ni—1.56 atomic percentage; B—2.20 atomic percentage (the rest is silicon and oxygen from the silica support). Similar values were obtained for the catalyst after the HDS/HDN process (Figure 3b), with 1.42 atomic percentage for Ni and 1.39 atomic percentage for boron, respectively. Furthermore, for the catalyst after the HDS/HDN process, the atomic percentage of element C as a carbon deposit was calculated at 18.20 atomic percentage. Relatively large atomic percentages of nickel on the catalyst surface point to the high dispersion of the active phase on the support’ surface. This is in line with the high CO chemisorption capability and a relatively high specific surface area of the catalyst (Section 3.2 and Table 1).

**Figure 3:**
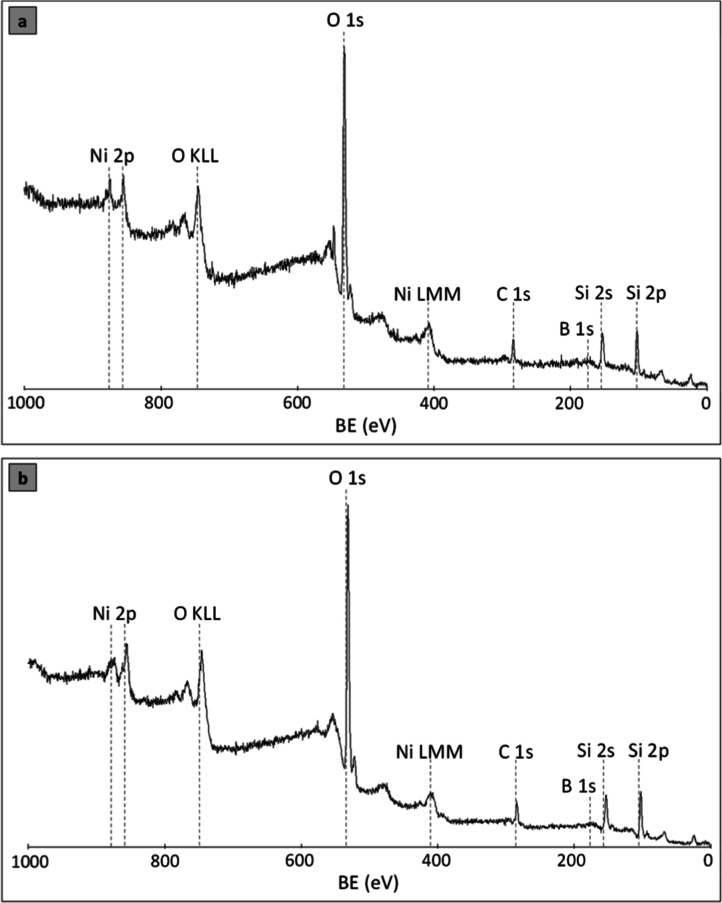
The XPS spectra for (a) reduced NiB/SiO_2_ and (b) spent NiB/SiO_2_ (after HDS/HDN).

The peaks of B 1s peaks with electron binding energies (BE) at 193.1 and 192.2 eV in both H_2_-treated and spent NiB/SiO_2_ catalysts, respectively, can be assigned to oxidized boron.^38^ First, during the process of catalyst preparation by the chemical reduction method, the occurrence of BH_4_^–^ hydrolysis toward oxidized B species was unavoidable. In addition, the oxidation of the elemental boron by oxygen in the air takes place when the samples are transferred from the reactor after reduction and catalytic tests. The intensities of the characteristic boron peaks are very small due to the fact that NiB does not constitute the entire mass of the catalyst but is deposited on the SiO_2_ material. Nevertheless, the XRD studies (Figure 2b) clearly show that the NiB phase is present in the NiB/SiO_2_ catalyst.

As seen in Figure 3, typical signals of oxidized boron (BE at 193.1 eV^38^) and zerovalent boron atoms involved in NiB (BE at 189.9–190.3 eV^23^) were detected on the surface of both reduced and spent catalysts (the B 1s spectrum is depicted in Figure S3). This is consistent with the XRD studies (Figure 2b), which clearly show the NiB phase present in the NiB/SiO_2_ catalyst.

Regarding Ni species in both samples, the Ni 2p envelope (Figure 4) could be deconvoluted into five spectral lines. As illustrated in Figure 4a, the XPS spectrum of the H_2_-treated sample presents two main signals centered at BE values of 856.3 and 874.6 eV assigned to the spin–orbit splitting of Ni 2p_3/2_ and Ni 2p_1/2_, respectively. The wide signals appearing around 862.8 and 866.3 eV correspond to the Ni 2p_1/2_ and Ni 2p_3/2_ shakeup satellites. The BE values strongly suggest the presence of oxidized nickel species, with a dominant contribution of Ni^2+^ moieties.^39,40^ For the H_2_-treated catalyst, apart from the mentioned signals, a small peak at 852.9 eV has appeared. It corresponds to the metallic Ni state, for which Ni (2p_3/2_) binding energies of 852.2 to 853.0 eV have been reported in supported forms.^41,42^

**Figure 4:**
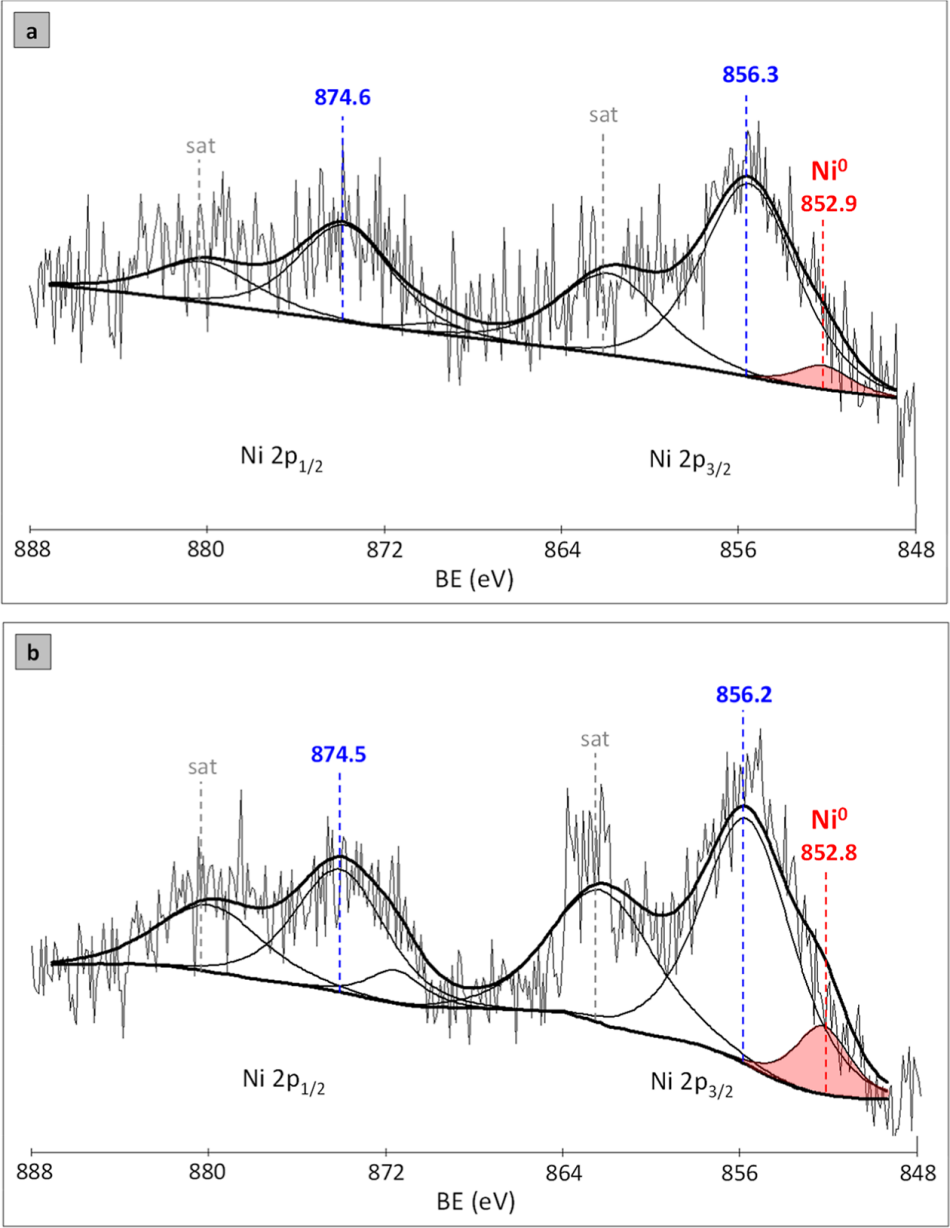
The XPS spectra of the Ni 2p region for (a) H_2_-treated (2 h, 450 °C) NiB/SiO_2_ and (b) spent (after 100 h of HDS/HDN) NiB/SiO_2_ samples.

The Ni 2p of the spent NiB/SiO_2_ catalyst (Figure 4b) also showed five components, that is, subpeaks at around 856.2 and 874.5 eV (with satellite peaks at 862.8 and 867.7 eV) and the small band at about 852.8 eV. In the case of this sample, the two major peaks can also be attributed to 2p_3/2_ and 2p_1/2_ of Ni^2+^ in the Ni–S phase (probably Ni_3_S_2_).^10,43^ That would be in agreement with the XRD measurements (Figure 2d) and with the FT-IR results (Figure 5), which have confirmed the presence of sulfur species on a spent NiB/SiO_2_ catalyst (cf. Section 3.6). It can also be seen that the corresponding position of each peak has changed slightly to lower BE values (within the error of the method) compared to those observed for the reduced sample. This probably indicates a small enhancement of the interaction between the carrier and active phase.

**Figure 5:**
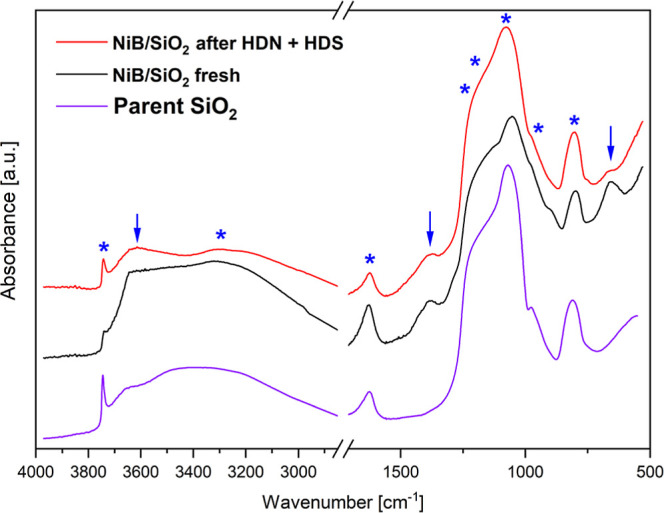
DRIFT spectra collected for parent SiO_2_, fresh NiB/SiO_2_, and the same catalyst after HDN and HDS (the characteristic silica and NiB bands marked by asterisks and arrows, respectively).

The poor degree of nickel species reduction should also be mentioned. For both H_2_-treated and spent NiB/SiO_2_ catalysts, the results show that the peak area of the elemental nickel (Ni^0^) signal is smaller than the signals of other Ni-containing moieties, indicating that only a minor fraction of nickel oxide was reduced. Since the sample reduction prior to XPS measurements involved the ex situ procedure, the effect of Ni species reducibility is insignificant. We can therefore anticipate that after the catalyst was removed from the reactor (upon the reduction process and cooling to RT), it has been superficially oxidized immediately after it came into contact with air. This issue is described in detail in the Supporting Information.

Although a loop was used to transfer the sample to the XPS instrument, under contact with atmospheric oxygen, it has been partially oxidized, and therefore only a small peak of Ni^0^ is visible in the XPS spectrum.

Here, the authors felt obliged to add a comment on the XPS results. Since the energy resolution of the XPS apparatus used hardly allowed for the correct distribution of Ni signals, only the listed species were taken into account. In addition to the major photoelectron peaks, our Ni XPS plots (Figure 4) feature broad shakeup satellites, where the degree of overlap in the respective envelopes is severe because these peaks have been broadly overlaid with each other. Therefore, the above analysis of nickel species should be considered semiquantitative.^44^

### 3.6 FT-IR Spectroscopy

Figure 5 shows the DRIFT spectra of the parent SiO_2_, and fresh and spent (after HDN and HDS) NiB/SiO_2_ material. As expected, these spectra feature a set of characteristic absorption bands for both components of the catalyst.

The modes of silica support vibrations are the following: (i) sharp band at 3745 cm^–1^ attributed to isolated silanol –OH stretchings; (ii) broad band at 3000–3700 cm^–1^ ascribed to the stretchings of hydrogen-bonded silanols; (iii) three intense, partially overlapping bands at 1240, 1205, and 1080 cm^–1^ ascribed to the asymmetric stretching mode of Si–O–Si and dehydroxylated superficial siloxane bridges; (iv) band at 970 cm^–1^ attributed to the stretching vibrations of Si–OH species; and (v) band at 805 cm^–1^ assigned to the Si–O–Si bending modes. Furthermore, both catalysts show the presence of a relatively sharp band at 1625 cm^–1^ assigned to the H–O–H bending mode of strongly physisorbed water. The active phase of the catalyst contributes to the spectra with two further absorption modes, namely, (i) the band of a minor intensity centered at 1380 cm^–1^ and (ii) the absorption mode at 658 cm^–1^. Partial surface oxidation takes place during catalyst synthesis and its removal from the reactor after the catalytic run to form oxygenated species of boron. So, band ∼1380 cm^–1^ is assigned to BO_3_ moieties of the stretch of the B–O bond, and ∼658 cm^–1^ is attributed to the vibrations of the B–O–B framework.^45^

It is pertinent to mention that the HDN and HDS processes alter the surface chemistry of both the silica support and the active phases. Namely, the silica gel underwent significant dehydration, which is clearly manifested by the increase in the intensity of the band at 3745 cm^–1^ accompanied by a pronounced decrease in the intensity of the bands at 3000–3700 cm^–1^ and 1625 cm^–1^. Particularly interesting is the new band emerging at 3615 cm^–1^ for the spent catalyst. This absorption mode can be ascribed to the presence of −SH surface groups as postulated formerly by Topsøe and Topsøe^46^ and Olivas et al.^47^

### 3.7 Catalytic Activity

#### 3.7.1 Hydrodesulfurization of 4,6-DMDBT

To inquire about the effect of the described properties of the NiB/SiO_2_ catalyst on its activity, HDS experiments of 4,6-DMDBT alone and HDS experiments of 4,6-DMDBT in the presence of carbazole were performed. Figure 6 presents the relation between conversion (or HDS degree) and the contact time (*t*_c_) for the HDS of 4,6-DMDBT reaction and the plots of ln­(1/1 – *x*) vs *t*_c_. The results (Figure 6a) explicitly demonstrate that for both test reactions, the difference between 4,6-DMDBT conversion and S removal rate equaled about 5% over the entire range of *t*_c_.

**Figure 6:**
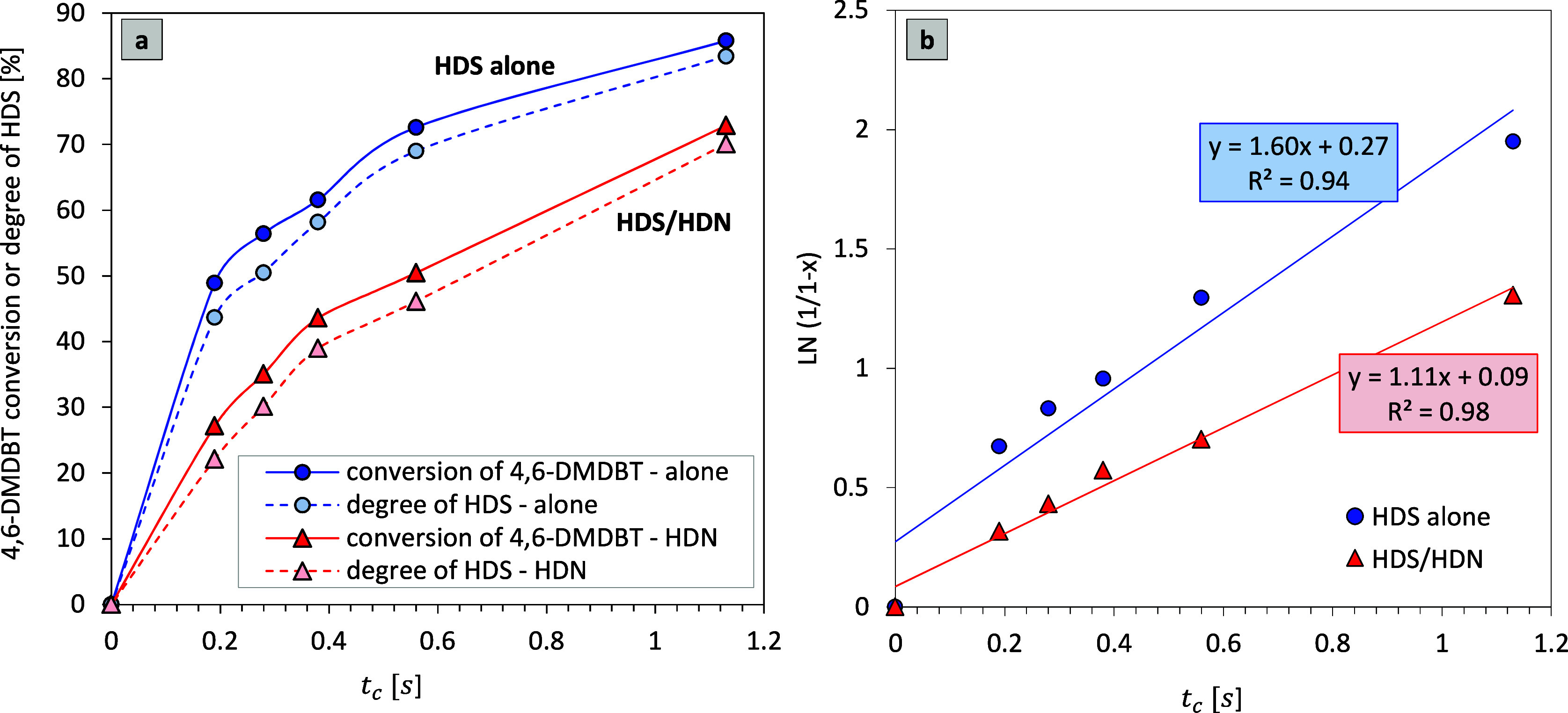
Catalytic performance of the NiB/SiO_2_ catalyst: (a) 4,6-DMDBT conversion (or HDS degree) (350 °C, 6.0 MPa H_2_, *t*_c_ = 0.19–1.13 s) and (b) pseudo-first-order kinetics linear plots for 4,6-DMDBT HDS conversion; ln­(1/1 – *x*) vs *t*_c_, where *x* means the 4,6-DMDBT conversion and *k*_4,6‑DMDBT_ is the overall pseudo-first-order rate constant for HDS of 4,6-DMDBT.

The presence of the nitrogen compound has a substantial influence on the conversion of 4,6-DMDBT. Figure 6a evidently shows that the simultaneous HDN of the carbazole process (as a competitive reaction) reduces the conversion and the S removal rate roughly by 15–20% compared to the HDS process conducted alone in a whole range of *t*_c_. The conversion of 4,6-DMDBT alterates with rising contact time (*t*_c_) from about 49 to 86% in the case of the alone HDS test, while only from 27 to 73% for the HDS/HDN experiment. The highest difference is noted for the contact time of 0.56 s. It is also noticeably visible in the influence of *t*_c_ on the conversion, e.g., to obtain the 50% conversion in the HDS alone test, a *t*_c_ of at least 0.19 s is required, while in the HDS/HDN experiment, this conversion is achieved for 0.56 s. The HDS reaction is found to be of a pseudo-first-order order with regard to 4,6-DMDBT in both catalytic tests (Figure 6b). The rate constant calculated for the HDS of 4,6-DMDBT carried out without carbazole is 1.5-fold higher than that for the catalytic test in the presence of an N-containing compound (Table 4).

**Table 4:**
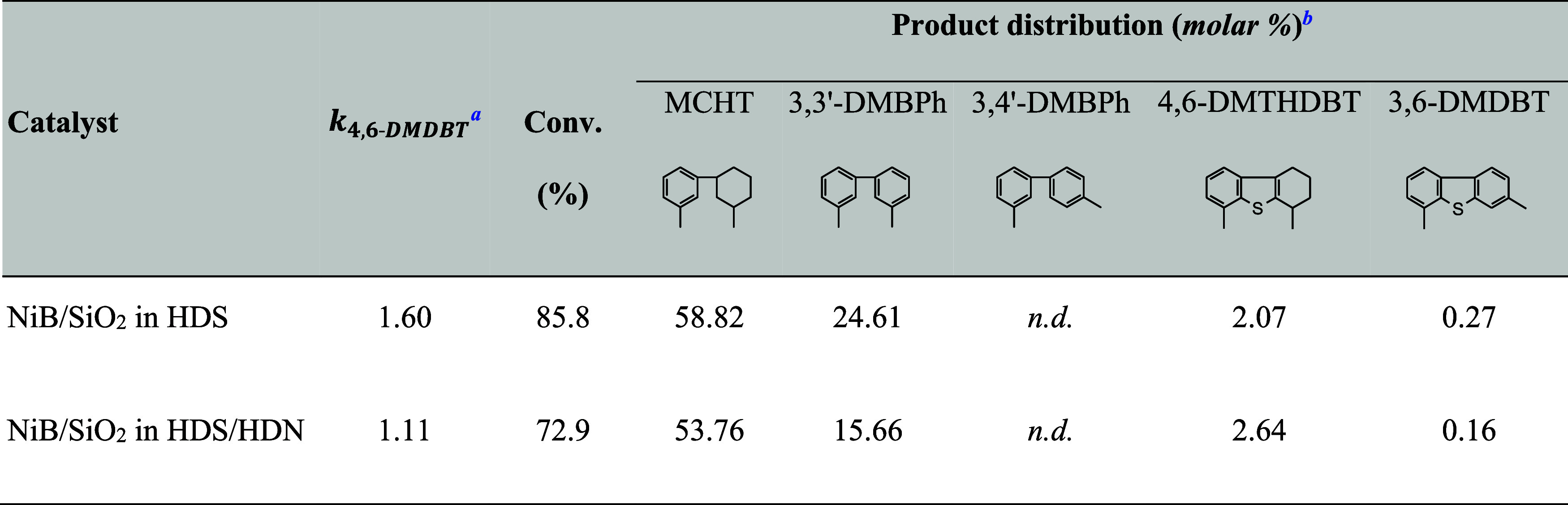
Catalytic Activity of NiB/SiO_2_ Catalysts in HDS of 4,6-DMDBT

a *k*_4,6‑DMDBT_ overall pseudo-first-order rate constant for HDS of 4,6-DMDBT.

b Product distribution at the highest 4,6-DMDBT conversion; n.d., not detected.

Taken together, these results indicate that the inhibiting effect (competitive reaction) of the simultaneous HDN reaction was substantial in lowering the HDS activity of the catalyst. Moreover, in both cases, even for the longest contact time (*t*_c_ = 1.13 s), the conversion and the HDS degree did not reach 100%. Therefore, the inhibitory effect of the parallel HDN of carbazole occurred over the entire range of the tested contact time.

Table 4 shows the distribution of products at the highest conversion obtained in each test, while Figure 7 shows the distribution of products over the entire range of contact times *t*_c_. Analysis of the reaction routes was carried out considering 4,6-dimethyltetrahydrodibenzothiophene (4,6-DMTHDBT) and 3-(3′methylcyclohexyl)-toluene (MCHT) as HYD pathway products and all dimethylbiphenyl isomers (mainly dimethylbiphenyl; 3,3′-DMBPh) as direct desulfurization (DDS) products. The conversion of 4,6-DMDBT leads primarily to the formation of hydrogenated HYD compounds (Figure 7). Regarding the distribution of the product over NiB/SiO_2_, for each *t*_c_ and regardless of the experiment mode, MCHT appeared as the major component among the reaction products (Table 4). The MCHT efficiency in the HDS test catalyst is higher than that in HDS/HDN, and it falls in the ranges of 30.6–58.8 mol % and 14.8–53.5 mol %, respectively. During the reaction, a small amount of 4,6-DMTHDBT intermediate is formed, and a maximum yield of approximately 5 mol % is obtained for a *t*_c_ of 0.19 s in the HDS process alone (Figure 7a) and for a *t*_c_ of 0.28 s in the case of parallel HDS/HDN reactions (Figure 7b). It should be noted that among all the products in the HDS alone and HDS/HDN experiments, 3,3′-dimethylbicyclohexyl (3,3′-DMBCH) is not found as a product of complete hydrogenation (Figure 7). In general, only small amounts of side-reaction products are observed under the tested conditions. The comparison of the variations in the efficiency of the DDS product, i.e., 3,3′-DMBPh, between these two experiments has revealed that there is a substantial decrease in the yield of 3,3′-DMBPh when the activity test is conducted with the accompanying nitrogen compound. The results demonstrate that the HDS and HDS/HDN catalytic tests provide the efficiency of the DDS product, that is, 3,3-DMBPh, of 13.1–24.6 and 5.7–15.7 mol %, respectively. In summary, all the findings indicate that the HYD pathway is the favored HDS reaction route, regardless of the nitrogen compound’s presence. Furthermore, the variations between the HDS and HDS/HDN tests in the efficiency of MCHT and 3,3′-DMBPh indicate that the inhibitory effect of the parallel HDN carbazole reaction affects DDS more strongly than it does on the HYD pathway.

**Figure 7:**
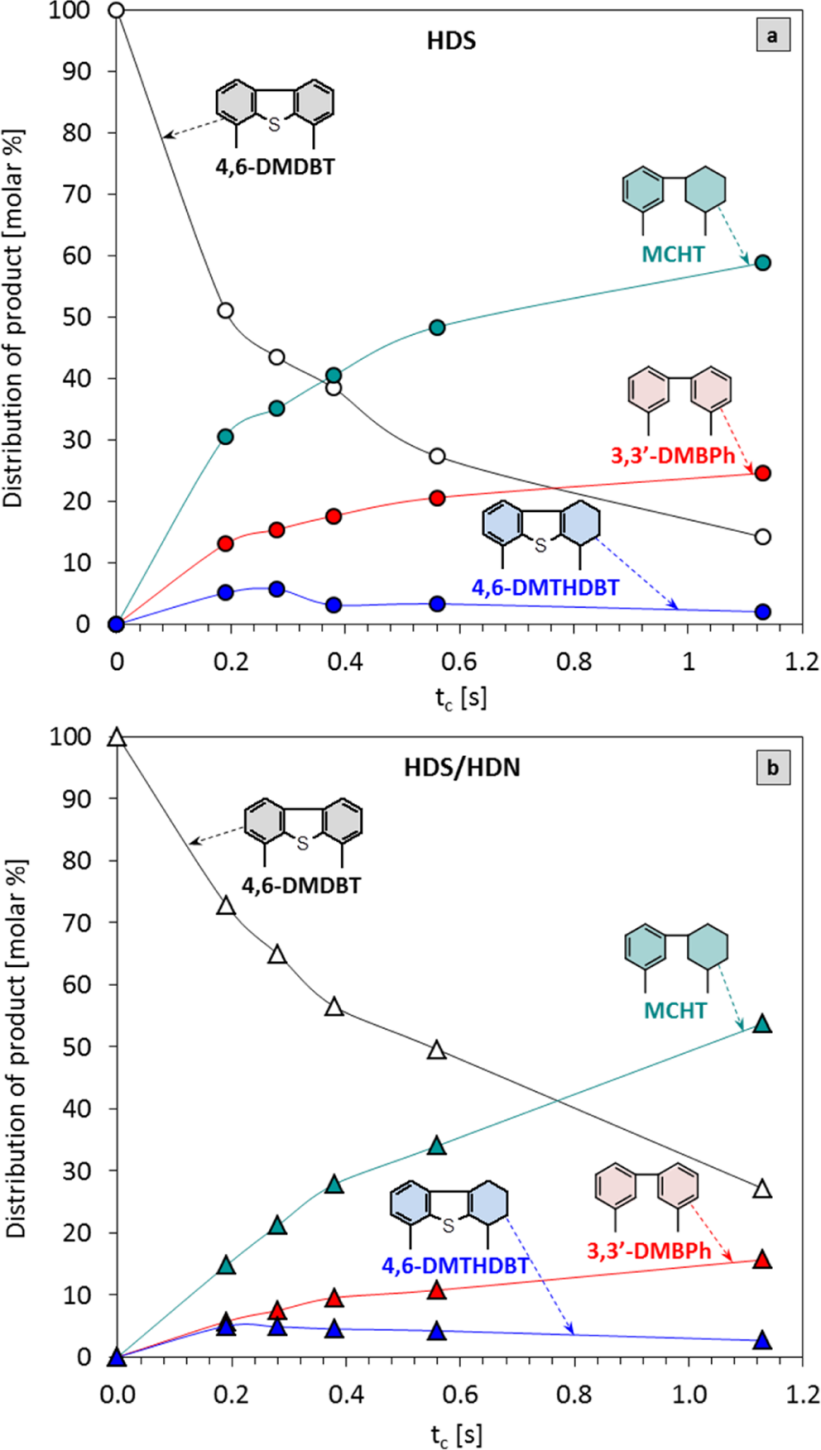
Product distribution for (a) the HDS of 4,6-DMDBT alone and (b) the HDS of 4,6-DMDBT with HDN.

#### 3.7.2 Stability of the Catalyst in HDS

Long-term stability tests of the NiB/SiO_2_ catalyst were performed for about 100 h on stream and the results are shown in Figure 8. The long-operating test includes the time of the actual catalytic experiment (determining the influence of *t*_c_ in the range 0.19–1.13 s), which was carried out for about 75 h.

**Figure 8:**
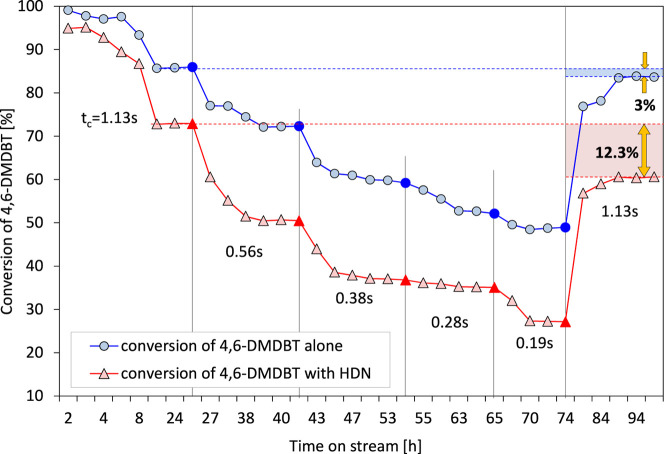
Multihour stability test over the NiB/SiO_2_ catalyst in the HDS of 4,6-DMDBT alone and together with HDN of carbazole; the range of *t*_c_ within 0.19–1.13 s.

After this time, the initial reaction conditions have been set back to those initial ones (i.e., the *t*_c_ of 1.13 s has been set), and the reaction has been continued for the next 20 h. It should be noted that the reaction was carried out under the specified conditions until a constant conversion was achieved. This includes the so-called stabilization of the NiB/SiO_2_ catalyst concept. These results are adopted as a measure of the catalyst stability by comparing 4,6-DMDBT conversion at the beginning (more precisely, after stabilization, that is, after 25 h of catalyst operation) and at the end of the test. The plots depicted in Figure 8 demonstrate that in the case of the catalytic HDS process conducted alone, the final 4,6-DMDBT conversion is almost equal to the one reached initially. Moreover, when 4,6-DMDBT is transformed alone, only slight deviations in conversion within *t*_c_ intervals are detected. Thus, it seems that under such conditions, the NiB/SiO_2_ catalyst reaches equilibrium relatively quickly. An entirely different phenomenon is observed in the experiment carried out with the 4,6-DMDBT/carbazole mixture. The stability of the NiB/SiO_2_ catalyst under such reaction conditions is no longer so visible. The noticeable decline in 4,6-DMDBT conversion because of the presence of nitrogen compounds may attain up to 12.3% (the comparison of the initial and final 4,6-DMDBT conversion in the HDS/HDN test). Also, the contribution of carbazole to catalyst stabilization steps is harmful due to the pronounced diminution in the 4,6-DMDBT conversion within the *t*_c_ intervals. Furthermore, for *t*_c_ below 1.13 s, the 4,6-DMDBT conversion in the HDS/HDN test is even 20% lower than that in the HDS alone one, which should be attributed to the hindering role of the nitrogen-containing molecule. Altogether, the results clearly show the strong inhibiting impact of the competitive HDN carbazole reaction on the conversion of 4,6-DMDBT.

Table 5 presents the comparative conversion results and degree of hydrodesulfurization (HDS) for the tested boride catalyst NiB/SiO_2_ and the nickel catalyst Ni/SiO_2_ with the same (10 wt %) nickel content. The results show that the NiB/SiO_2_ catalyst was more active than the nickel catalyst. The greatest differences in activity (S removal rate and conversion) were observed for short contact times (0.19–0.38 s). In both cases, the tests were carried out for about 100 h.

**Table 5:** The Results of HDS over NiB/SiO_2_ and Ni/SiO_2_ Catalysts

	NiB/SiO_2_		Ni/SiO_2_	
contact time [s]	S removal rate (%)	HDS (%)	conv. (%)	HDS (%)
1.13	72.9	70.1	68.2	66.4
0.56	50.5	46.1	46.7	43.6
0.38	36.8	32.1	29.7	26.9
0.28	35.1	30.2	25.1	22.5
0.19	27.2	22.1	20.9	16.7

## 4 Discussion

The results show that the presence of carbazole exerts a strong influence on the HDS of the 4,6-DMDBT reaction conducted over a NiB/SiO_2_ catalyst. Therefore, let us consider the features of the NiB/SiO_2_ catalyst in order to explain its catalytic performance in HDS and HDS/HDN experiments.

### 4.1 Surface Active Species

A significant issue in this research is the chemistry of the NiB/SiO_2_ catalyst surface in view of the identification of the active species involved in the reaction. Figure 2 shows a series of XRD diffractograms of the SiO_2_ support and NiB/SiO_2_ fresh, pretreated, and spent samples. The results revealed that all of the patterns contain broad reflection, which is characteristic of the amorphous SiO_2_ support (at 2θ = 25°). The XRD spectrum of the fresh NiB/SiO_2_ catalyst (Figure 2b) shows the typical wide diffraction line for the amorphous NiB phase (2θ = 45°).^38,48^ After annealing of the NiB/SiO_2_ catalyst at 350 °C (i.e., at the reaction temperature) under a hydrogen atmosphere, in the XRD spectrum (Figure 2c), two new peaks appeared which are representative for metallic nickel (at 2θ = 44.3° and about 51.6°). The third characteristic peak of Ni^0^ (for 2θ at about 76.1°) is barely visible due to its low intensity. This proves that the NiB supported in the SiO_2_ alloy segregates under the influence of an elevated temperature and hydrogen into metallic nickel and well-dispersed boron, invisible in the spectrum. The same Figure 2 also shows the XRD pattern (Figure 2d) of the catalyst after the HDS/HDN reaction, with characteristic peaks attributed to the formation of Ni_3_S_2_. Thus, the catalyst inevitably undergoes sulfidation during the HDS and HDS/HDN reactions. However, analysis of the elemental sulfur content (Table 2) in the catalyst after the HDS/HDN process shows that only 54.3% of nickel is converted to Ni_3_S_2_, which is also corroborated by XRD and XPS results. Therefore, it is essential to consider the thermodynamic conditions and their influence on the equilibrium of the Ni vs Ni_3_S_2_ system, as the presence of such a sulfide phase was found based on the XRD pattern. However, it should be noted that the HDS (HDS/HDN) process over NiB/SiO_2_ in our investigation was not preceded by a classical sulfidation procedure (10% H_2_S in H_2_) as used in the work by Skrabalak and Suslick.^17^ They have demonstrated that the boron phase is one of the contributors of active species after the sulfide atom of nickel boride. According to the fundamental work of Kirkpatrick,^49^ at the reaction temperature of 350 °C, the value of the H_2_S/H_2_ ratio corresponding to the Ni/Ni_3_S_2_ equilibrium equals 1.43 × 10^–5^. Below this value, nickel can exist in the catalyst in its metallic phase. Furthermore, H_2_S chemisorption is an exothermic reaction, therefore removal of sulfur from the surface will be favored by an increase in temperature.^50^ Also, the chemisorption of H_2_S on the nickel surface is reversible and depends on the *p*_H_2_S_/*p*_H_2__ ratio. According to the thermodynamic data,^50–52^ the surface of nickel saturation was observed within the temperature range of 550–645 °C for a *p*_H_2_S_/*p*_H_2__ ratio of 2–5 × 10^–6^, where a bulk Ni_3_S_2_ was formed under a *p*_H_2_S_/*p*_H_2__ ratio of about 10^–3^ to 10^–4^. When the model HDS reaction is conducted in the range of different contact times (*t*_c_ from 0.19 to 1.13 s), the conversion of the sulfur compound changes simultaneously, causing changes in the H_2_S partial pressure. And, naturally, the value of the H_2_S partial pressure affects the degree of sulfidation of the active nickel phase. The activity results of the 4,6-DMDBT transformation show that the reached conversion varies from about 49 to 87% and 28 to 61% for the HDS alone process and combined HDS/HDN, respectively. Thus, the *p*_H_2_S_/*p*_H_2__ ratio (H_2_/feed = 600) varies in the range of 2.94 × 10^–4^ to 8.23 × 10^–5^, which lies roughly in the limiting range of the *p*_H_2_S_/*p*_H_2__ partial pressure ratio. This suggests that sulfidation of the active species takes place only to some extent. Bezverkhyy et al. in their research^53,54^ hypothesized a more complex mechanism of nickel catalyst sulfidation. The authors have unexpectedly found that Ni sulfidation is incomplete, despite the high partial pressure of thiophene provided.^54^ This phenomenon is explained by the two-step mechanism of nickel sulfidation. In the first stage, thiophene undergoes the HDS reaction, and then the resulting H_2_S reacts with the metallic Ni. In the second stage, after sulfidation, the catalytic activity of the metal can change, causing the partial pressure of H_2_S to change during the reaction. As catalyst sulfidation progresses, the HDS activity and H_2_S partial pressure decrease. Eventually, H_2_S may reach equilibrium with the Ni/Ni_3_S_2_ system, leading to partial sulfidation of Ni and a steady-state at a moderate transformation level.^54^ Therefore, it can be assumed that during the HDS reaction, the NiB species in the NiB/SiO_2_ catalyst initially undergoes the same process, i.e., decomposition into Ni and well-dispersed boron. However, at the same time, the presence of a sulfur compound such as 4,6-DMDBT leads to partial sulfidation of the catalyst, as shown by the XRD spectrum of the spent catalyst (Figure 2d), where characteristic peaks originating from Ni_3_S_2_ are detected. Bezverkhyy and co-workers^53,54^ have inferred that the interaction of the sulfur compound does not cause direct sulfidation of the Ni/SiO_2_ catalyst. However it cannot be ruled out that the thermodynamic conditions of the Ni/Ni_3_S_2_/H_2_/H_2_S system cause a shift of the equilibrium toward metallic nickel. As mentioned above, the relative stabilities of the sulfides vary with reaction conditions.^53,55^ Under the HDS atmosphere involving the presence of H_2_ and H_2_S in the gas phase, in the chemical equilibrium expressed by eq 4, the sulfided Ni particles should be reduced according to the reaction:^55^

4 $${Ni}_{3}S_{2}+{xH}_{2}↔{Ni}_{3}S_{2-x}+{xH}_{2}S$$

On the other hand, the reduction of bulk Ni_3_S_2_ into metallic nickel can occur as the lowest limit of sulfur’s chemical potential according to chemical equilibrium ([Disp-formula eq5]):^55^

5 $${Ni}_{3}S_{2}+{2H}_{2}↔3Ni+{2H}_{2}S$$

As inferred above, the main cause of incomplete sulfidation of the NiB/SiO_2_ catalyst was the processing variables, but it cannot be ruled out that the thermodynamic conditions of the Ni/Ni_3_S_2_/H_2_/H_2_S system also played a significant role. The equilibrium between Ni and sulfur species depends on the reaction temperature, the H_2_/feed ratio, and hydrogen pressure, that is generally based on the hydrotreating process conditions, but also on the resistance of nickel (due to the presence of boron) to the poisonous effect of sulfur.^18–20,56^ Bartholomew and Uken^56^ found that nickel boride and Raney nickel catalysts show much higher sulfur resistance. This is due to a lower deactivation rate and higher sulfur adsorption capacity, attributed to electronic modification of the nickel surface by electron transfer from boron. According to the theoretical study by Luo et al.,^18^ sulfur atoms prefer to bond to boron instead of nickel in the NiB alloy catalyst. This leads to protection of the active nickel from deactivation by sulfur. These findings are fully consistent with those previously reported by Li et al.^19^ and Wang et al.^20^

### 4.2 Hydrodesulfurization

As mentioned above, the test reaction of 4,6-DMDBT over the NiB/SiO_2_ catalyst was carried out in two modes, i.e., without the presence of carbazole (HDS) and with its presence (HDS/HDN). It is well-known that N-compounds have an inhibitory influence on the HDS process.^57^ Additionally, our results (Figure 6) clearly imply that the addition of carbazole leads to a significant decrease in HDS conversion over that of the NiB/SiO_2_ catalyst. This is especially evident in the long-term performance of the catalyst (Figure 8). Considering the competitive adsorption of N- and S-compounds, almost all the adsorption energies of N-compounds and their intermediates are higher than those of S-compounds with similar structures.^57^ In principle, not only the N-compounds themselves act as harmful molecules, but the actual competitive adsorption inhibitor may be partially or fully saturated HDN intermediates as well as a byproduct, i.e., ammonia. Hence, as the reaction proceeds, the concentration of nitrogen-containing intermediates in the reaction medium is higher, thus negatively affecting the performance of the catalyst.

The activity test demonstrates that without an N-compound in the feedstock, the NiB/SiO_2_ catalyst exhibits higher activities than in the case of the combined HDS/HDN process, with respective maximal conversions of 86 and 73%, respectively (Table 4). The results also reveal that the HYD route in the desulfurization of 4,6-DMDBT is dominant and is less inhibited by carbazole than the DDS route. For example, at the highest conversion (Table 4), the yield of HYD products in the HDS/HDN test is lower by approximately 5% compared to the HDS one, while the yield of DDS products is lower even by 9%. However, the reaction, irrespective of the presence of carbazole, followed two reaction paths, i.e., the DDS route giving 3,3′-DMBPh as a product and the HYD route yielding MCHT as the main product (Figure 7 and Scheme 1). Hence, the reaction proceeded classically as for sulfide or metallic catalysts.^58–60^

**Scheme 1:**
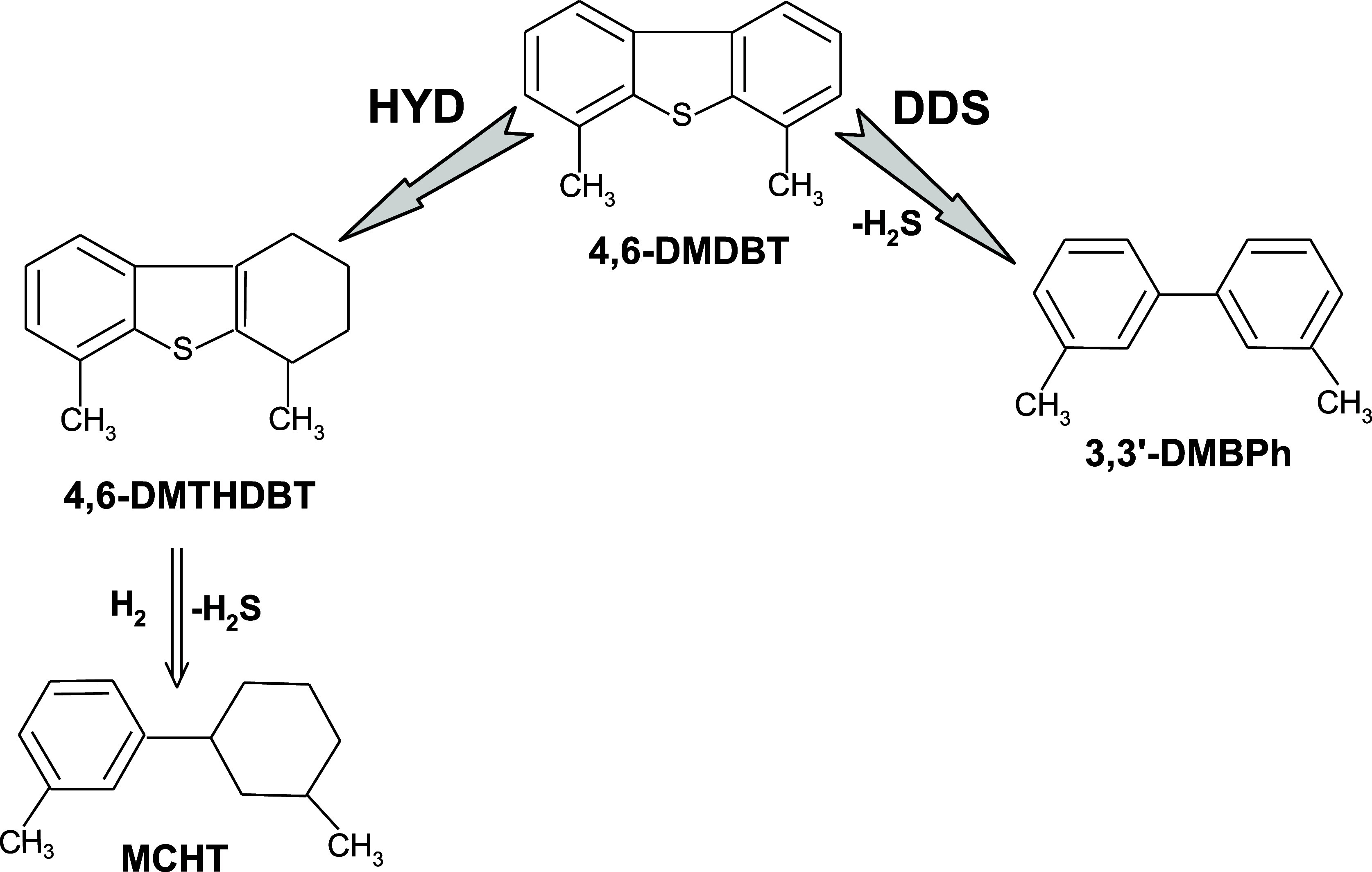
Pathways of the HDS of 4,6-DMDBT Reaction over the Supported NiB/SiO_2_ Catalyst

Interestingly, among all products, no fully hydrogenated compound was observed, i.e., 3,3′-dimethylbicyclohexyl (3,3′-DMBCH). This molecule can be formed during the deep hydrogenation of 4,6-DMDBT to perhydro-4,6-DMDBT, which is directly desulfurized to MCHT. The other possibility is to hydrogenate MCHT; however, hydrogenation of the aromatic ring in MCHT is difficult. The structure of the MCHT molecule is not flat, which impedes its adsorption on the catalyst surface.^58^ In our case, the amount of MCHT increases markedly with an increasing contact time. Hence, it can be assumed that MCHT is formed directly by the desulfurization of hexahydro-4,6-DMDBT. Similarly, the lack of 3,3′-DMBCH in the HDS reaction products was noticed by other authors^59,60^ who used precious metals as the active phase. Similarly, in the work dealing with the Ni_2_P catalyst, it was found that DMBPh was not further hydrogenated to 3,3′-DMBCH under the same reaction conditions as in our investigation.^6^

Regarding the decomposition of products into NiB/SiO_2_, the main desulfurized product obtained over this catalyst is MCHT, indicating that the HYD route is the dominant one in the HDS reaction. These results are consistent with our previous studies of nonsupported NiB alloys.^21^ The favored pathway for the HDS reaction determines the entire cost of the process and governs the quality of the product. On the one hand, the DDS pathway is considered cost-effective because it consumes a lower amount of H_2_ than the HYD pathway. On the other hand, the HYD pathway provides the component with a less aromatic character and, as a consequence, with higher quality. The pathway of the HDS reaction of 4,6-DMDBT, one of the possible routes, depends on the manner of interaction of the 4,6-DMDBT molecule with the active site. As stated in the studies,^61^ the activation of the DDS pathway of sulfur in the thiophenic ring is initiated by adsorbing the sulfur atom on the active sites, it forms a σ bond with them, leading to hydrogenolysis of the C–S bond. The formation of a σ bond between the sulfur atom of thiophenic acid and the catalytic site is possible mainly on the electron-deficient center, where the S atom with a lone electron pair can be easily adsorbed. This indicates that electron deficiency in the active site is selectively beneficial for the direct desulfurization (DDS) pathway. The formation of the σ-bond between the sulfur atom of 4,6-DMDBT and the active species faces steric hindrance arising from the presence of the alkyl group neighboring the S atom. Because of that, the adsorption of 4,6-DMDBT likely occurs planar via the engagement of the electrons of the aromatic rings. A strong bond between the metal and the sulfur compound can form^4^ (by the C=C bond) and^5^ entities (by delocalized electrons). Therefore, if the electron density of the active site is sufficient, it forms a π bond with the aromatic ring of the sulfur compound and therefore the C–C bond of the aromatic ring is first saturated and then the C–S bond is broken, that is, according to the HYD route. In this regard, it is not surprising that the catalytic performance of the NiB/SiO_2_ catalyst may be affected by the increase in the electron density (active phase) of the catalyst caused by the presence of B species, which favors the HYD pathway. Under the steady state of the NiB/SiO_2_ catalyst operating under the conditions investigated in this work, the active surface consists of two phases, that is, metallic nickel (also assisted by boron) and the Ni_3_S_2_ species (constituted during the reaction progress as a result of Ni sulfidation). Nickel species are commonly accepted to be active in hydrogenation reactions.^62^ In the NiB alloy, the occupancy of 3d nickel orbitals is more privileged than that for pure nickel, and the 3d position is important for the catalysis of hydrogenation. Hybridization of Ni spd states with B sp ones increases its resistance to poisoning of NiB by sulfur.^63^ Following the suggestion presented in ref^64^, the high HYD activity observed for the NiB/SiO_2_ catalyst might also be clarified considering that the produced H_2_S influences the equilibrium between DDS and HYD active sites, inhibiting the DDS route far stronger than the HYD one. This is due to the significant competition of H_2_S with 4,6-DMDBT for adsorption at the vacant sites, leading to the inhibition of the DDS pathway. However, further experiments are necessary to confirm and explain this idea in detail. Moreover, the adsorption of a sulfur compound is stronger on a metallic Ni atom than on its sulfide.

A key issue of the HDS catalyst is its deactivation under processing conditions and the negative influence of ‘poisoning’ molecules contained in the feed or byproducts of the reaction. The metallic Ni formed by the decomposition of the NiB phase is exposed to further transformations under the influence of the ongoing reaction. Consequently, Ni is exposed to deactivation caused by adverse effects of sulfur compounds. In the case of nickel catalysts, the problem is their relatively high sensitivity to the presence of sulfur due to the strong adsorption of H_2_S on their surfaces.^50,52^ The degree of sulfidation of the catalyst and the degree of its ‘poisoning’ depend on the reaction conditions.^53,65^ Moreover, the structure of the catalyst surface, as well as its electronic properties, plays a role in maintaining its resistance to sulfur poisoning.^66^

Under the reaction conditions (the temperature 350 °C, hydrogen pressure, and the presence of compounds derived from the decomposition of the feed molecules) the sulfidation undergoes to form nickel sulfide species (in this work Ni_3_S_2_) as well. As mentioned above, the elemental analysis shows that 53.4% of Ni was converted to the Ni_3_S_2_ phase. It was reported elsewhere that Ni and Co sulfides deposited on an inert carbon support (in this work, SiO_2_ can be considered an inert support) show higher activity than MoS_2_/C.^67^ Furthermore, Neurock and van Santen^68^ have postulated that the Ni_3_S_2_ cluster is relatively active in the HDS reaction. Therefore, it can be assumed that not only is the metallic nickel accompanied by boron active in the HDS reaction, but also the Ni_3_S_2_ species deposited on the inert SiO_2_ support are involved as well. In the case of the HDS alone experiment (Figure 8), the conversion of 4,6-DMDBT has dropped by about 3% (the conversion comparison reached at *t*_c_ = 1.13 s in the first phase of the process and the final conversion of 4,6-DMDBT in the HDS test). Therefore, it can be expected that the resulting Ni_3_S_2_ (which is formed rather later in the reaction) also shows certain HDS activity, albeit lower than that of the metallic nickel phase, which existed only on the catalyst surface in the initial hours of the process, where the 4,6-DMDBT conversion was close to 100%.

For both HDS of 4,6-DMDBT and HDN of carbazole reactions, hydrogenation is the first stage of the reaction, and it can take place on the same active centers of the catalyst.^69^ The high activity of the NiB/SiO_2_ catalyst, along with the maintained high HYD activity, may also be attributed to the species newly formed under the reaction conditions. According to Neurock and van Santen,^68^ hydrogen adsorbs molecularly and heterolytically dissociates on Ni_3_S_2_, forming both adsorbed sulfhydryl (SH) and hydryl (MeH) forms. The catalytic sites for this dissociation consist of a sulfur vacancy located on the metal atom and an adjacent sulfur anion. The hydride-type H atom is adsorbed on the metal ion, while the protonic-type H atom is anchored on the sulfur anion, forming SH moieties in this way. This confirms the hypothesis of Topsøe and Topsøe,^46^ who postulated that sulfhydryl (SH) groups are formed on the catalyst surface as a result of H_2_ dissociation and that they are responsible for the hydrogenation activity of thiophene derivatives. The phenomenon of SH and MeH species formation was also suggested by several authors.^64,70,71^ Indeed, on the basis of FT-IR results for the spent catalyst (Figure 5), it was evidenced that the −SH groups were formed during the operation of the NiB/SiO_2_ catalyst. Hence, one might suppose that the catalytic behavior of the NiB/SiO_2_ system is also due to the formation of specific centers for the heterolytic dissociation of H_2_ on its surface. However, further research is needed to confirm this supposition.

To summarize, the results clearly illustrate that the HDS of the 4,6-DMDBT reaction over the NiB/SiO_2_ catalyst, regardless of the presence of carbazole, takes place mainly in the HYD route on metallic nickel species (assisted by boron species) featuring a strong hydrogenation functionality. It is well-known that the acidity of the catalyst has an influence on the hydrogenation activity—acid centers take an active part in hydrogen activation—especially Brønsted acid centers. This is also confirmed by acidity measurements (3.94 μmolNH_3_/g for HDS reaction alone and 2.89 μmol NH_3_/g for HDS/HDN reactions), which may indicate the presence of Brønsted acid centers in the form of S–H groups. Olivas et al.^47^ suggested the formation of surface acidic S–H groups as a result of joining the nickel sulfide sulfur atom with a hydrogen atom. Thus, the increase in selectivity of the boride catalyst in the HYD path can also be attributed to an increase in catalyst acidity as a result of the presence of boron on the catalyst. Furthermore, the high catalytic activity may arise from the high degree of nickel boride dispersion on the support surface. This finds reflection in the relatively high specific surface area (Table 1) and high CO chemisorption capacity (Section 3.2). However, the NiB/SiO_2_ catalyst also shows activity in the DDS route. This activity can be attributed to nickel sulfide clusters, i.e., sulfur vacancy (coordinately unsaturated sites, CUS).^64^ Furthermore, the HDS reaction over a NiB/SiO_2_ catalyst is feasible under such conditions to prevent complete sulfidation of the catalyst. Finally, it was found that boron present on the surface of the catalyst plays a major role in its resistance to sulfur poisoning. To summarize, the NiB/SiO_2_ catalytic system offers several fundamental benefits that surpass the commercially employed catalysts. These advantages are as follows:^8,9,15,18,20–22^

(i) NiB supported on mesoporous SiO_2_ features high hydrogenating performance desirable in deep hydrotreating,
(ii) it shows higher activity than only Ni-based catalysts, including Raney nickel,
(iii) the NiB is characterized by a higher resistance to poisoning by sulfur which usually causes deactivation of classical catalysts,
(iv) the nickel boride catalysts are substantially cheaper compared to the noble metals-based ones.

## 5 Conclusions

In this work, we studied the HDS of 4,6-DMDBT by using a NiB/SiO_2_ catalyst. The presented work was carried out to investigate the effect of the SiO_2_ support on the activity of the NiB catalyst as well as to elucidate the effect of carbazole on the HDS reaction carried out on the studied catalyst. The results of these investigations can be summarized as follows:

- NiB deposited in SiO_2_ showed activity in the HDS 4,6-DMDBT reaction. The main product of the reaction carried out alone and in the presence of a nitrogen compound (carbazole) was MCHT (formed along the HYD path). With much lower selectivity, HDS from the 4,6-DMDBT process followed the DDS route and formed 3,3′-DMBPh as a product. The fully hydrogenated 3,3′-dimethylbicyclohexyl (3,3′-DMBCH) product among the HDS reaction products was not observed.
- Present research shows that during the HDS reaction, metallic nickel and Ni_3_S_2_ are formed on the catalyst surface. However, the main activity of the catalyst is attributed to the presence of the zerovalent nickel, accompanied by highly dispersed boron species. The presence of a competitive carbazole HDN reaction strongly (negatively) influenced the conversion of 4,6-DMDBT in the HDS reaction on NiB/SiO_2_. A detrimental effect of the N-compound is more pronounced in the DDS pathway.
- Under appropriate conditions, it is possible to carry out the HDS 4,6-DMDBT reaction (deep HDS) on a nickel–metal phase catalyst.
- The Ni_3_S_2_ phase formed during the process is also the active phase in the HDS/HDN reaction under study.

## Supplementary Material



## Notes

Additional data regarding the 10%Ni/SiO_2_ reference catalyst in the form of the XRD spectrum of a reduced Ni/SiO_2_ catalyst (the catalyst being first reduced at a temperature of 450 °C and under hydrogen flow), the XPS spectrum of the Ni 2p region for reduced Ni/SiO_2_ sample, and the XPS spectrum of the B 1s region for spent NiB/SiO_2_ sample after HDS/HDN reactions (PDF)
