# Correction to “Activity of Amorphous NiB/SiO_2_ in Hydrotreating Model Reactions: Hydrodesulfurization of 4,6-DMDBT”

**DOI:** 10.1021/acsomega.6c10341

**Published:** 2026-09-24

**Authors:** Marek Lewandowski, Rafał Janus, Mariusz Wądrzyk, Karolina Jaroszewska, Kamila Zaborowska

The authors regret that Figure 2b in the original article contains an incorrect XRD data set. During figure preparation, a composite diffraction pattern consisting of diffraction data recorded separately for the pristine SiO_2_ support (10–90° 2θ) and bulk NiB (38–50° 2θ) was inadvertently plotted in place of the experimental pattern for the supported NiB/SiO_2_ catalyst. To ensure full transparency and maintain the accuracy of the published article, the corrected version of Figure 2 is provided below, presenting the recorded XRD pattern of the supported NiB/SiO_2_ catalyst. Importantly, the authors wish to emphasize that this correction does not affect the interpretation or discussion of the results and has no impact on the findings or conclusions of the article.

**Figure 2:**
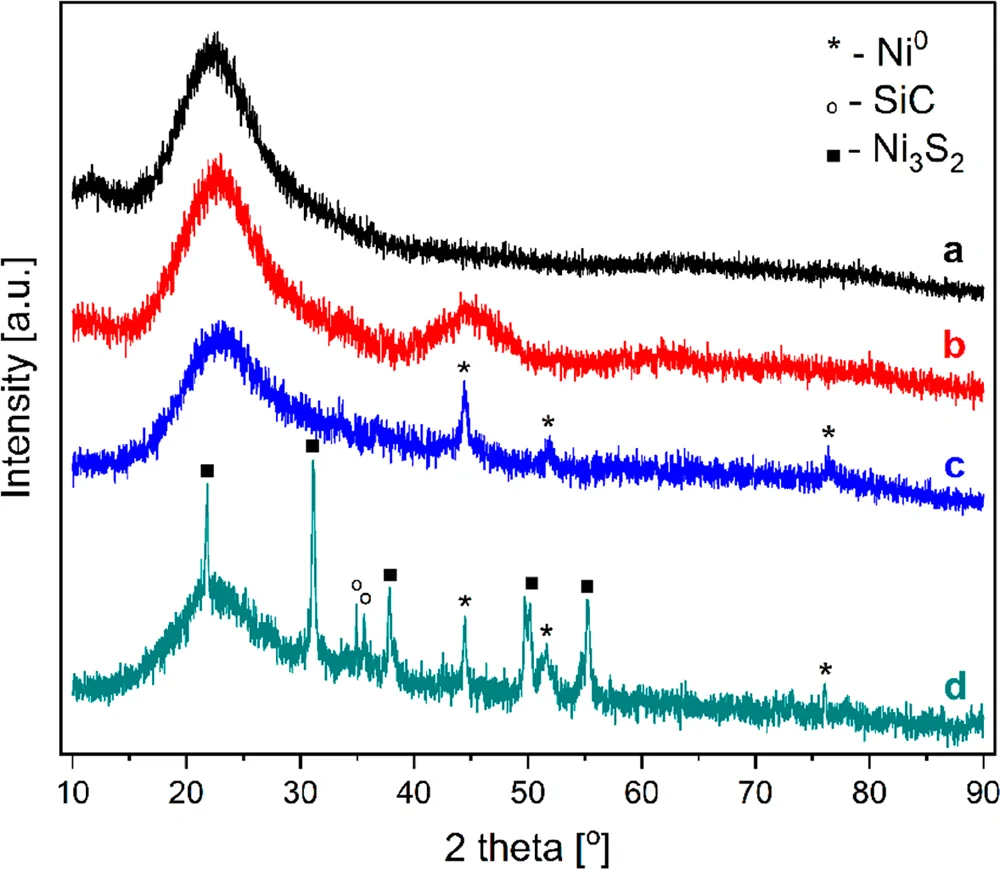
X-ray diffraction patterns of: (a) pristine SiO_2_ support, (b) fresh NiB/SiO_2_ catalyst, (c) NiB/SiO_2_ catalyst heated in H_2_ for 8 h at 350 °C, and (d) NiB/SiO_2_ catalyst after HDS/HDN reaction of 4,6-DMDBT.

