# Supplementary material for: Activity of Amorphous NiB/SiO2 in Hydrotreating Model Reactions: Hydrodesulfurization of 4,6-DMDBT
Source: ACS Omega. 2025 Aug 20;10(34):38968–84. doi: 10.1021/acsomega.5c04966 (PMC12412260; doi:10.1021/acsomega.5c04966)
Supplement: Supplementary file 1 [file ao5c04966_si_002.pdf]

Activity of amorphous NiB/SiO_2 in hydrotreating model reactions - hydrodesulfurization of 4,6-DMDBT

Marek Lewandowski[a,*], Rafał Janus[a], Mariusz Wądrzyk[a], Karolina Jaroszewska[b], Kamila Zaborowska[a].

AUTHOR INFORMATION

[a] *AGH University of Krakow, Faculty of Energy and Fuels, 30 Mickiewicza, PL-30059 Kraków, Poland*

[b] *Wrocław University of Science and Technology, Faculty of Chemistry, 7/9 Gdańska, PL-50344, Wrocław, Poland*

*E-mail: lewandowski@agh.edu.pl

ORCID

Marek Lewandowski: 0000-0002-4204-6352

A number of our works concerned the hydrotreating properties of molybdenum and tungsten carbide. Due to their pyrophoric, their passivation was inevitable as one of the elements of catalyst preparation. We have had cases in our experience where the rapid removal of the reduced catalyst caused it to heat up strongly as a result of oxidation also Ni. In the case of a nickel catalyst (10% Ni/SiO_2), reduction and passivation *in-situ* (in quartz reactor without removal of the catalyst). The catalyst was first reduced at a temperature of 450°C and under hydrogen flow. After cooling the quartz reactor to room temperature, the catalyst was passivated for few hours by pulsed technique (*in situ*) in separate apparatus (He as a carrier). Then, after removal of the catalyst from the reactor, XRD and XPS measurements were performed on the catalyst sample as shown below.

The presented XRD spectrum (Fig. S1) shows distinct reflections corresponding to metallic nickel. On the other hand, the XPS analysis of the same catalyst sample (Fig.S2), similarly to the NiB/SiO_2 catalyst (also content 10% of Ni) being the subject of the publication, mainly the presence of oxidized nickel Ni^{2+} with a residual amount of surface metallic nickel on the surface. So, there was no deep oxidation of the nickel. It should be borne in mind that the XPS analysis reaches the depth of only about 10 nm, i.e., it is a surface method. XRD analyzes are analyzes covering much greater depths. The problem of surface oxidation of the reduced catalysts is a difficult one. Being aware of this, the procedure for removal of the catalyst from the reactor was based something like “passivation" as follows: after cooling the reactor to room

temperature under a hydrogen atmosphere, the inlet and outlet of the reactor (1/8 inch Swagelok) were purposely loosened to create a small leak in some sense. This situation allowed for a very slow drop in H_2 pressure and slow diffusion of air. The reactor was left in this state for about 24 h and only then the catalyst was removed from the reactor tube. Therefore, we believe that in our case no deep oxidation took place, because the presence of reflections from metallic nickel Ni^0 was observed in the XRD spectrum. On the other hand, the XPS spectra concern much smaller depths of surface research - as mentioned earlier. However, trace amounts of Ni^0 can be observed on the spent catalyst sample and plus Ni^0 present in the XRD spectrum allowed us to conclude that while the catalyst is operating in a stable reactor, Ni^0 is present as the active phase. And the XPS spectrum in Figure S2 is very similar to the XPS spectrum in Figure 4 in the manuscript.

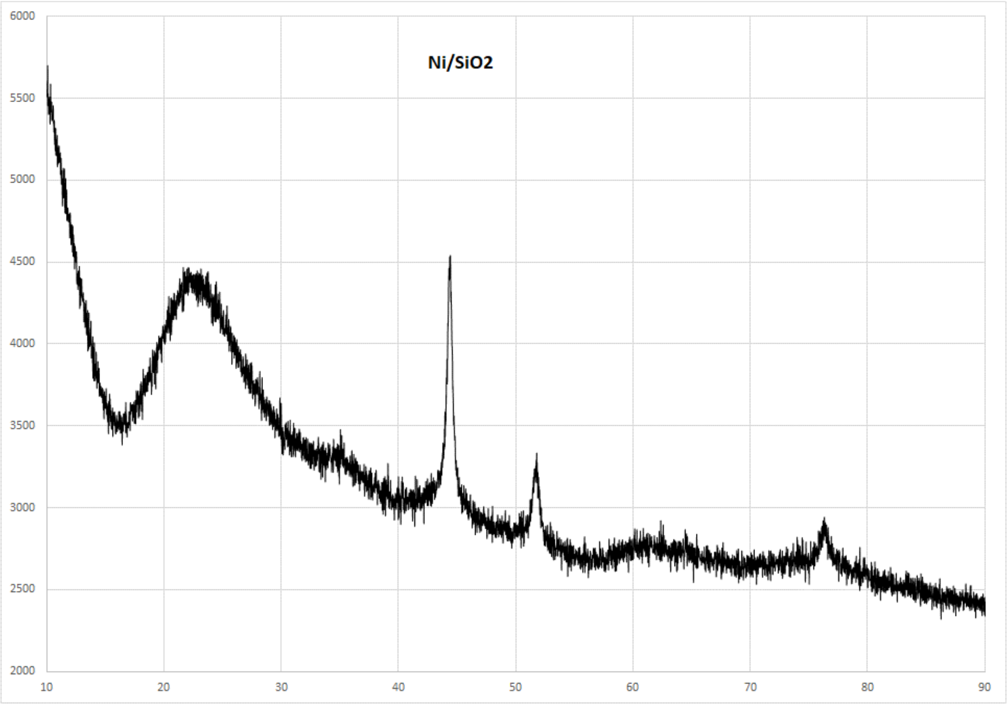

Figure S1. XRD spectrum of Ni/SiO_2 catalyst.

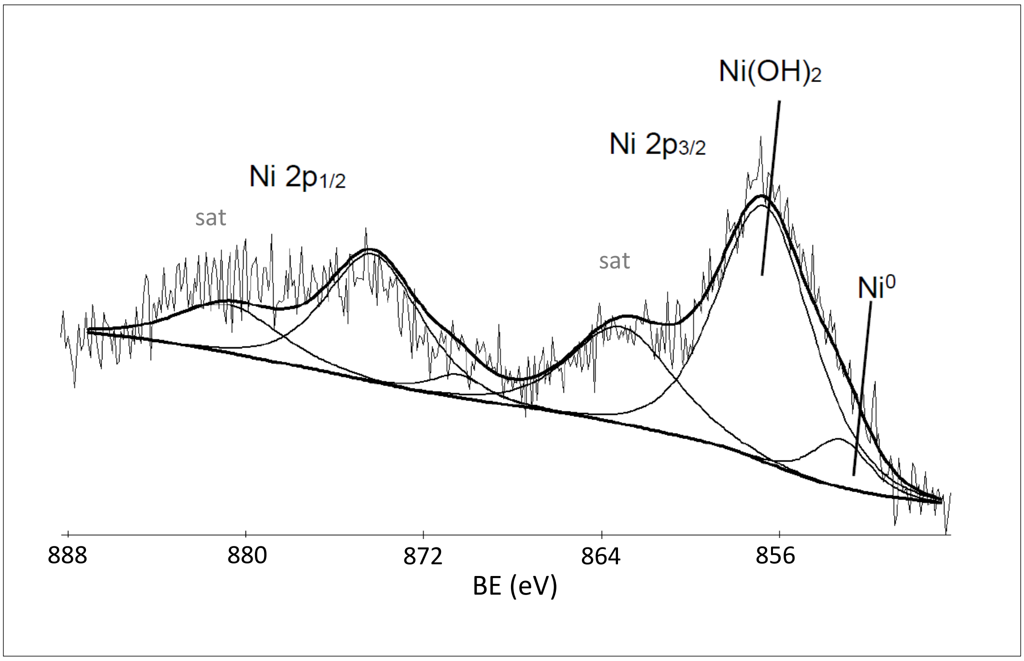

Figure S2. The XPS spectrum of Ni 2p region for reduced Ni/SiO_2 sample.

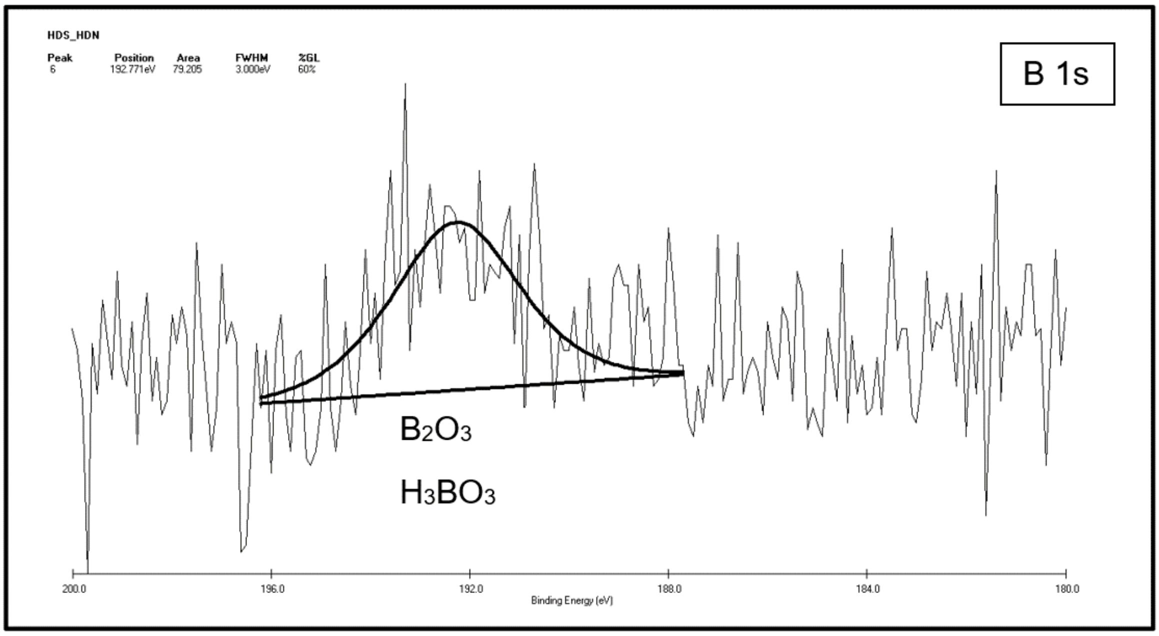

Figure S3. The XPS spectrum of B1s region for NiB/SiO_2 sample after HDS/HDN reactions.
